# High-affinity chromodomains engineered for improved detection of histone methylation and enhanced CRISPR-based gene repression

**DOI:** 10.1038/s41467-022-34269-7

**Published:** 2022-11-15

**Authors:** G. Veggiani, R. Villaseñor, G. D. Martyn, J. Q. Tang, M. W. Krone, J. Gu, C. Chen, M. L. Waters, K. H. Pearce, T. Baubec, S. S. Sidhu

**Affiliations:** 1 The Anvil Institute, Kitchener, ON N2G 1H6 Canada; 2 Department of Pathobiological Sciences, School of Veterinary Medicine, Louisiana State University, Baton Rouge, LA 70803 USA; 3 Division of Molecular Biology, Biomedical Center Munich, Ludwig-Maximilians-University, 82152 Planegg-Martinsried, Germany; 4 Department of Molecular Mechanisms of Disease, University of Zurich, 8057 Zurich, Switzerland; 5 School of Pharmacy, University of Waterloo, Waterloo, ON N2L 3G1 Canada; 6 Department of Chemistry, University of North Carolina at Chapel Hill, CB 3290, Chapel Hill, NC 27599 USA; 7 Center for Integrative Chemical Biology and Drug Discovery, Division of Chemical Biology and Medicinal Chemistry, UNC Eshelman School of Pharmacy, University of North Carolina at Chapel Hill, Chapel Hill, NC 27599 USA; 8 Division of Genome Biology and Epigenetics, Institute of Biodynamics and Biocomplexity, Department of Biology, Utrecht University, 3584 Utrecht, The Netherlands

**Keywords:** Methylation analysis, Genetic engineering

## Abstract

Histone methylation is an important post-translational modification that plays a crucial role in regulating cellular functions, and its dysregulation is implicated in cancer and developmental defects. Therefore, systematic characterization of histone methylation is necessary to elucidate complex biological processes, identify biomarkers, and ultimately, enable drug discovery. Studying histone methylation relies on the use of antibodies, but these suffer from lot-to-lot variation, are costly, and cannot be used in live cells. Chromatin-modification reader domains are potential affinity reagents for methylated histones, but their application is limited by their modest affinities. We used phage display to identify key residues that greatly enhance the affinities of Cbx chromodomains for methylated histone marks and develop a general strategy for enhancing the affinity of chromodomains of the human Cbx protein family. Our strategy allows us to develop powerful probes for genome-wide binding analysis and live-cell imaging. Furthermore, we use optimized chromodomains to develop extremely potent CRISPR-based repressors for tailored gene silencing. Our results highlight the power of engineered chromodomains for analyzing protein interaction networks involving chromatin and represent a modular platform for efficient gene silencing.

## Abstract (web summary)

Engineered chromodomains are promising probes to analyze histone methylation. Here the authors designed high-affinity chromodomains for enhanced genome-wide binding analysis, live-cell imaging and ultra-potent CRISPR-based gene repression.

## Introduction

Post-translational modifications (PTMs) are covalent modifications of peptides and proteins that expand and diversify protein function beyond their coding genome^1^. PTMs are essential regulators of protein conformation, localization, stability, abundance, activity and interactions^2^, and their dysregulation has been linked to a variety of diseases, including cancer, diabetes, and neurodegeneration^3^.

Protein methylation is one of the most abundant PTMs in prokaryotes and eukaryotes^4^. In particular, lysine methylation has been extensively investigated in the context of histone N-terminal tails, where methylation mainly functions as a signal for the recruitment of effector proteins that contribute to chromatin remodeling^5^. Histone methylation can occur at several arginine and lysine residues on histones, resulting in a vast and dynamically different array of signals that regulate transcription, DNA replication, DNA repair, and cell cycle control^6^. More recently, methylation of non-histone proteins has emerged as a key regulator in a variety of cellular pathways^7,8^, further highlighting the relevance of such PTMs for cell physiology. Indeed, aberrant protein methylation has been described in a large variety of human pathologies^3,9^ but deciphering the functional role of lysine methylation remains a major challenge, due to the lack of robust technologies for probing and analyzing methylated proteins.

Antibodies that recognize methylated lysine residues suffer from low affinity, poor specificity and lot-to-lot variations^10^, thus limiting their utility for the analysis of methylation sites, particularly in live cells. As an alternative, several groups have exploited naturally occurring methyllysine-binding domains (reader domains) as tools for the detection of methyllysine-containing proteins^11–14^. However, the intrinsically weak affinity and specificity for discrete methylated states (e.g. mono-, di-, or tri-methylation) has limited the application of these natural domains^15,16^.

Chromodomains, the best-studied methyllysine readers, are modular domains of ~60 residues that are characterized by three β-strands packed against an α-helix^17^. The chromodomain fold includes an “aromatic cage” consisting of three aromatic amino acids supplemented by one or two acidic residues, which establish crucial cation–π interactions with the methyllysine ammonium group^18^. Despite chromodomains showing a high degree of structural conservation, they display significant differences in their binding preferences^19,20^, suggesting that different chromodomains could be used together for comprehensive methylome analysis and synthetic biology applications.

Several groups have engineered chromodomains and other epigenetic readers for mass spectrometry and chromatin enrichment^11,12^, as well as for the development of biosensors^21^, but the modest affinity of reader domains has limited their application. Only a few studies have focused on improving the binding affinity of epigenetic reader domains, and the majority of these efforts were directed at engineering HP1 chromodomains that naturally recognize H3K9me3 marks^22–25^. While these efforts yielded Cbx1 variants with enhanced affinity relative to the parental chromodomain and allowed mechanistic investigation of methyllysine recognition, they all targeted the H3K9me3 mark. Therefore, we aimed to develop a general approach that can be applied to a variety of histone modification reader domains.

Here we present a phage-displayed library to enhance the affinity of the chromodomain of Cbx7 for H3K27me3 marks, as well as a general method for enhancing the affinity of chromodomains of the Cbx protein family. Affinity selections and assays enabled us to identify two amino acid substitutions that substantially enhanced Cbx7 affinity for methyllysine-containing peptides without altering its specificity both in vitro and in living cells. Notably, these two substitutions also enhanced the affinities of many other human chromodomains, without altering their specificities. We further show that engineered Cbx chromodomains can be used as powerful probes for genome-wide binding analysis and live-cell imaging. Moreover, we used optimized Cbx chromodomains to develop highly potent CRISPR-based repressors for specific translational repression that significantly outperformed current gene silencing methods.

## Results

### Development of high-affinity variants of the Cbx7 chromodomain

To develop a chromodomain variant with enhanced affinity for lysine methylation, we focused on the chromodomain of human Cbx7 (Cbx7-chromo). We selected Cbx7-chromo over other human chromodomains for several reasons: (1) unlike Cbx1-chromo which is characterized by high affinity for H3K9me3, but does not bind to H3K27me3 peptides, Cbx7-chromo exhibits low affinity for both the H3K9me3 and H3K27me3 marks (*K*_D_ = 55 or 110 µM, respectively)^26^, (2) it lacks cysteine residues that can interfere with phage display, and (3) it showed strong display on phage in comparison with other chromodomains (Supplementary Fig. 1).

**Fig. 1:**
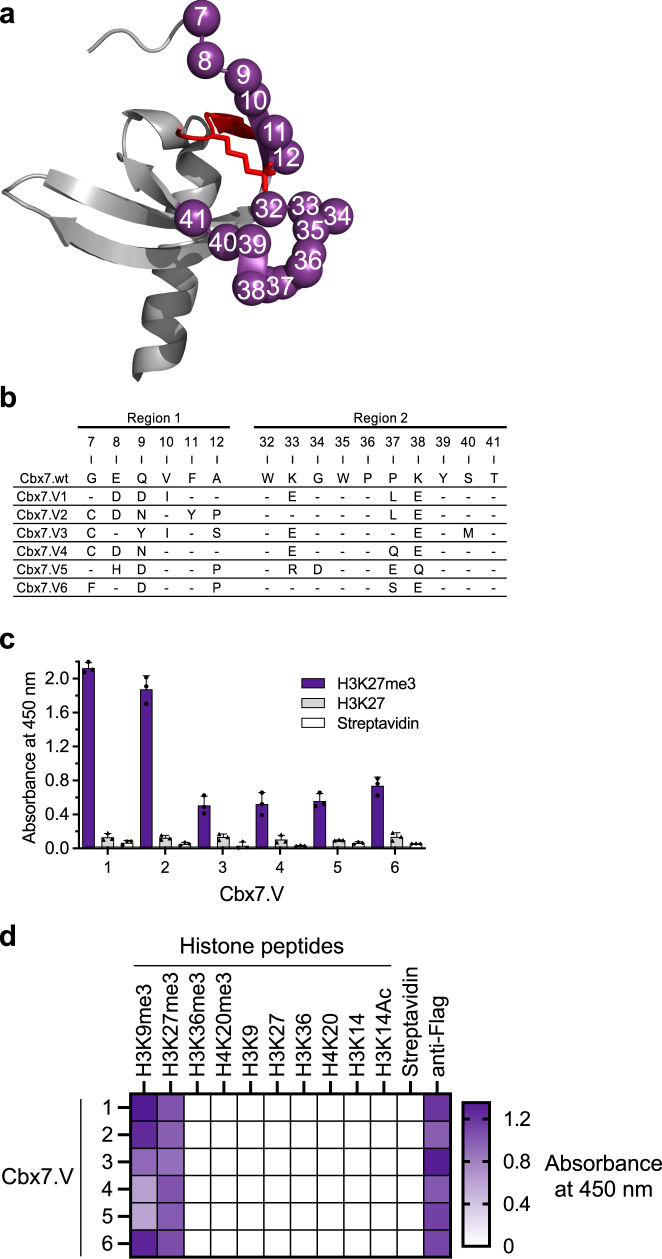
Design and characterization of optimized Cbx7 chromodomains. **a** Design of the phage-displayed library mapped onto the structure of Cbx7-chromo in complex with a H3K27me3 peptide (PDB code: 4X3K). The chromodomain is shown as a gray ribbon with diversified positions shown as purple spheres and numbered according to the sequence of full-length Cbx7 (UniProt ID: O95931). The H3K27me3 peptide is shown in red with the methyllysine side chain shown as sticks. **b** Sequence alignment of selected Cbx7.Vs. Only positions that were diversified in the library are shown, and dashes indicate residues that matched the wild-type sequence. **c** ELISAs for phage-displayed Cbx7.Vs binding to immobilized H3K27me3 peptide, H3K27 peptide or streptavidin. Data represent the mean and standard deviation of triplicate measurements. **d** Phage ELISAs for assessment of specificity of Flag-tagged Cbx7.Vs binding to immobilized histone peptides, streptavidin (negative control), or anti-Flag antibody (positive control). The heatmap shows the mean of absorbance values at 450 nm from three independent experiments in a purple gradient.

To aid library design, we examined the structure of Cbx7-chromo in complex with a H3K27me3 peptide (PDB: 4X3K)^27^, and we identified for diversification two continuous stretches that lined the methyllysine-binding pocket, which included 6 residues within the N-terminal β-strand (positions 7–12, region 1) and 10 residues on the other side of the pocket (positions 32–41, region 2; Fig. 1a). This residue set included three key amino acids that form the aromatic cage (Phe11, Trp32, and Trp35)^28^. These 16 positions were systematically subjected to a soft randomization mutagenesis strategy that favored the wild-type (wt) sequence but allowed ~50% mutations at each position^29^, and we constructed a phage-displayed library containing 1.1 × 10^10^ unique Cbx7-chromo variants (Cbx7.Vs).

The phage-displayed Cbx7.V library was cycled through five rounds of binding selections with an immobilized H3K27me3 peptide, and a negative selection step was included to deplete clones that bound to the non-methylated version of the H3K27 peptide. DNA sequencing of selected clones revealed six unique Cbx7 variants (Cbx7.V1-6, Fig. 1b), and most substitutions were found in seven positions (7, 8, 9, 12, 33, 37, and 38). The three positions that form the hydrophobic cage were conserved, except for a conservative F11Y substitution in one variant. Notably, the variants were all more negatively charged than the wild-type chromodomain, due to acidic substitutions in place of positively charged residues Lys33 and Lys38, and neutral residue Gln9. Phage ELISAs confirmed that all six variants bound to the H3K27me3 peptide, but not to the H3K27 peptide or streptavidin (Fig. 1c).

We assessed by phage ELISA the binding specificity of each Cbx7.V against a panel of tri-methylated and non-methylated histone peptides (see Supplementary Table 1 for sequences) and observed that all the variants bound only to H3K9me3 and H3K27me3 peptides (Fig. 1d). For further characterization, we focused on Cbx7.V1, because it exhibited the strongest signal for the H3K27me3 peptide by phage ELISA and it did not contain any cysteine residues. Isothermal titration calorimetry (ITC) assays showed that Cbx7.V1 bound to H3K9me3 and H3K27me3 peptides with near 1:1 stoichiometry and affinities in the low micromolar range (*K*_D_ = 7.0 and 7.8 µM for H3K9me3 or H3K27me3, respectively; Supplementary Fig. 2 and Supplementary Table 2).

### Site-directed mutagenesis of Cbx7.V1 and Cbx7.wt

Cbx7.V1 contains six substitutions relative to the wild-type chromodomain, and to investigate how each substitution affected function, we mutated each of the six residues individually back to the wild-type sequence and measured affinities for the H3K9me3 and H3K27me3 peptides using bio-layer interferometry (BLI, Table 1 and Supplementary Fig. 3). Only two back-mutations resulted in large reductions in affinity: D9Q and E33K reduced affinity for the H3K9me3 or H3K27me3 peptide, respectively. These results showed that negatively charged substitutions were mostly responsible for enhanced affinity, but surprisingly, the E38K back-mutation resulted in moderate increases in affinities for both peptides.

**Table 1:** Dissociation constants for Cbx7.V1 and back-mutants for methyllysine-containing peptides

Domain	*K*_D_ (µM)	
	H3K9me3	H3K27me3
Cbx7.V1	4.5 ± 0.5	2.0 ± 0.1
D8E	5.0 ± 0.5	2.0 ± 0.2
D9Q	>20	2 ± 1
I10V	3.5 ± 0.5	2.0 ± 0.2
E33K	10 ± 2	>20
L37P	3.0 ± 0.5	2.5 ± 0.5
E38K	2.0 ± 0.1	1.5 ± 0.1

Dissociation constants (*K*_D_) were determined by BLI for the chromodomains binding to immobilized H3K9me3 or H3K27me3 peptide. Results are mean of duplicate ±1 SD. See Supplementary Fig. 3 for BLI traces and Supplementary Table 1 for peptide sequences.

To further explore the effects of Asp9 and Glu33 residues in Cbx7.V1, we introduced these two substitutions either separately or together into wild-type Cbx7-chromo (Cbx7.wt) and measured affinities for the H3K9me3 and H3K27me3 peptides by fluorescence polarization (FP) (Table 2 and Supplementary Fig. 4). Both single substitutions improved affinities for both peptides, and consistent with the back-mutation analysis of Cbx7.V1 (Table 1), the Q9D or K33E substitution had the greatest effect on affinity for the H3K9me3 or H3K27me3 peptide, respectively. The variant containing the double Q9D/K33E substitutions (Cbx7.VD) exhibited higher affinity for both peptides compared with the variants with single substitutions, and these affinities were virtually identical to those of Cbx7.V1. Importantly, Cbx7.VD retained specificity for methyllysine, as it did not bind to unmethylated H3K9 or H3K27 peptides (Supplementary Fig. 5). Taken together, these data confirmed that, amongst the six substitutions in Cbx7.V1, the two substitutions Q9D/K33E are the only ones that contribute significantly to affinity enhancement for methylated histone peptides.

**Table 2:** Dissociation constants for Cbx7.wt and variants for methylated histone peptides

Domain	*K*_D_ (µM)	
	H3K9me3	H3K27me3
Cbx7.wt	30 ± 10	110 ± 40
Cbx7 Q9D	10 ± 1	100 ± 20
Cbx7 K33E	20 ± 3	20 ± 2
Cbx7.V1	7.0 ± 1.5	4.0 ± 0.5
Cbx7.VD	8.0 ± 0.5	5.0 ± 0.2

Dissociation constants (*K*_D_) were determined by fluorescence polarization for chromodomains binding to FITC-labeled H3K9me3 or H3K27me3 peptide. Results are mean of triplicate ±1 SD. See Supplementary Fig. 4 for binding curves and Supplementary Table 1 for peptide sequences.

### Effects of the Asp9/Glu33 double-substitution across the human Cbx chromodomain family

**Fig. 2:**
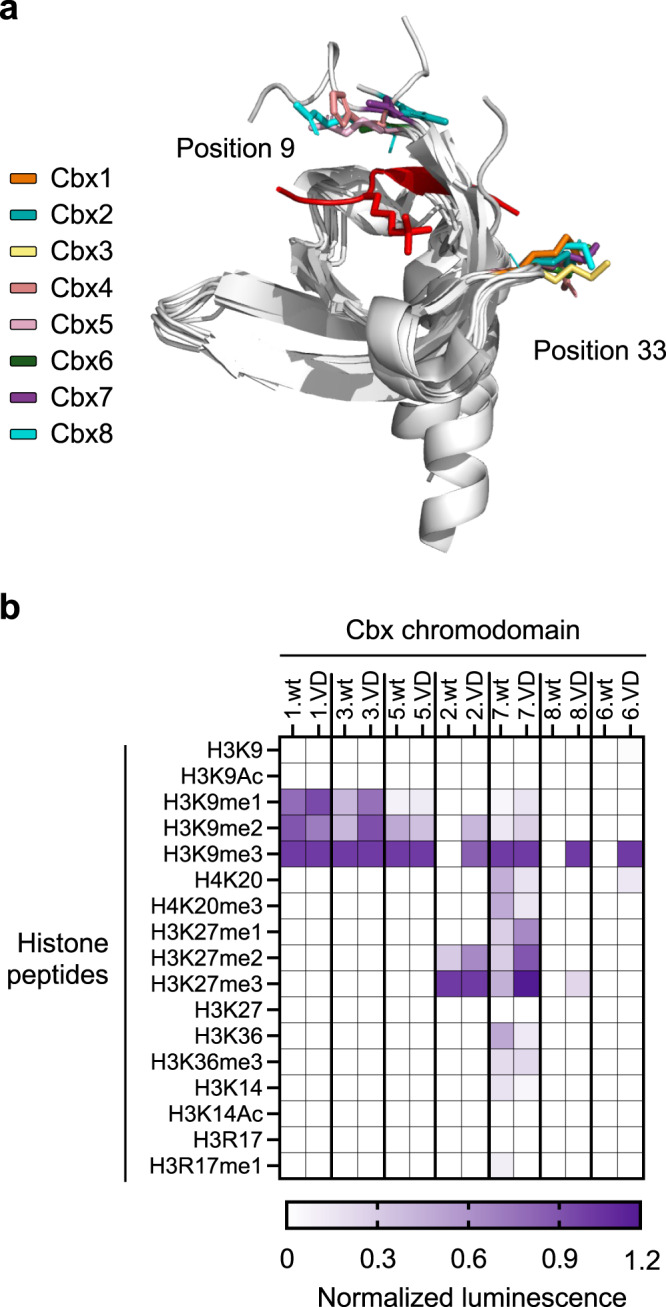
Specificities of wild-type and high-affinity chromodomains of PcG and HP1 family members. **a** Superposition of all human Cbx chromodomain structures with that of Cbx7 in complex with an H3K27me3 peptide (PDB: 4X3K). The H3K27me3 peptide is shown in red, with the methylated lysine residue shown as sticks. Mainchains of all domains are represented as gray ribbons, and sidechains of residues at positions 9 and 33 are shown as colored sticks (Cbx1, PDB: 3F2U; Cbx2, PDB: 3H91; Cbx3, PDB: 3DM1; Cbx4, PDB: 5EPL; Cbx5, PDB: 3FDT; Cbx6, PDB: 3I90; Cbx7, PDB: 4X3K; Cbx8, PDB: 3I91). Side chains at position 9 of Cbx1, Cbx3, and Cbx6 are missing, as they were not visible in the structures. **b** Specificity profiling of Cbx chromodomain-luciferase fusions with a panel of methylated and non-methylated histone peptides. Luminescence intensity for each domain was normalized to intensity for the H3K9me3 peptide, except for Cbx2.wt and Cbx2.VD, for which it was normalized to intensity for the H3K27me3 peptide. The mean of normalized luminescence intensity from three independent experiments is shown in a purple gradient.

We next explored whether the Asp9/Glu33 double-substitution could enhance the functions of other chromodomains from the polycomb group (PcG) of proteins (Cbx2, Cbx4, Cbx6, Cbx7, and Cbx8) and the heterochromatin protein 1 (HP1) family members (Cbx1, Cbx3, and Cbx5). We first investigated the primary sequence of each chromodomain and observed a conserved positively charged residue at position 33 (Supplementary Fig. 6a). While all PcG protein members contained polar or positively charged residues at position 9 (except for Cbx6, which contained a Glu), all HP1 family members were characterized by a Glu residue at the same position (Supplementary Fig. 6a). Next, we superposed the structures of all Cbx chromodomains with that of Cbx7-chromo in complex with an H3K27me3 peptide and mapped the location of residues at positions 9 and 33 (Fig. 2a and Supplementary Dataset 1). The other structures superposed well with that of Cbx7-chromo (RMSD = 0.5–2.0 Å), and in all cases, the side chains at positions 9 and 33 aligned well with those of Cbx7-chromo. Thus, we reasoned that, given the high structural similarity and high sequence homology of Cbx chromodomains, the Asp9/Glu33 substitutions may be able to enhance the affinity of the other Cbx chromodomains for methylated histone peptides.

To test this hypothesis, we substituted residues at positions 9 and 33 with Asp or Glu, respectively, in all Cbx chromodomains (Supplementary Dataset 2 and 3) and thus assembled a “Cbx.VD” panel (“VD” indicates the Asp9/Glu33 double-substitution). We used FP assays to measure the affinities of each wild-type Cbx chromodomain (Cbx.wt) and its Cbx.VD version for the H3K9me3 and H3K27me3 peptides (Table 3 and Supplementary Fig. 7). In the case of Cbx4-chromo, the substitutions had a detrimental effect, and they had no effect in the case of Cbx1-chromo, which already bound to the H3K9me3 peptide with sub-micromolar affinity. We previously enhanced the affinity of Cbx1-chromo via the introduction of a K33A mutation^22^, suggesting that the domain favors a hydrophobic histone-interacting surface rather than an electronegative one. Since the substitutions had a negative effect on Cbx4-chromo, we assessed the thermal stability of both Cbx4.wt and Cbx4.VD and found that the substitutions destabilized the domain (Supplementary Fig. 8). In all other cases, affinities were improved significantly. Like Cbx1-chromo, the chromodomains of Cbx3 and Cbx5 bound only the H3K9me3 peptide, and in both cases, the substitutions improved binding for this peptide but did not enhance binding to the H3K27me3 peptide. Like Cbx7-chromo, the chromodomains of Cbx2 and Cbx8 bound weakly to both peptides, and in both these cases, the substitutions dramatically improved binding to both peptides, as was the case for Cbx7-chromo. Finally, Cbx6-chromo did not bind appreciably to either peptide, but the Asp9/Glu33 double-substitution did enable binding to the H3K9me3 peptide.

**Table 3:** Dissociation constants for Cbx.wt and Cbx.VD pairs for methyllysine-containing peptides

Domain	*K*_D_ (µM)	
	H3K9me3	H3K27me3
Cbx4.wt	80 ± 10	>200
Cbx4.VD	NB	NB
Cbx1.wt	0.5 ± 0.1	NB
Cbx1.VD	0.6 ± 0.1	NB
Cbx3.wt	2 ± 1	NB
Cbx3.VD	0.35 ± 0.1	NB
Cbx5.wt	5.0 ± 0.5	NB
Cbx5.VD	0.5 ± 0.1	NB
Cbx2.wt	>200	100 ± 20
Cbx2.VD	5 ± 1	3.0 ± 0.2
Cbx7.wt	30 ± 10	110 ± 40
Cbx7.VD	8.0 ± 0.5	5.0 ± 0.2
Cbx8.wt	>200	>200
Cbx8.VD	20 ± 5	>50
Cbx6.wt	NB	NB
Cbx6.VD	>50	NB

Dissociation constants (*K*_D_) were determined by fluorescence polarization for the chromodomains binding to FITC-labeled H3K9me3 or H3K27me3 peptides. Results are mean of triplicate ±1 SD. “NB” indicates “no binding detected”. See Supplementary Fig. 7 for binding curves and Supplementary Table 1 for peptide sequences.

To gain structural insights about the effect of Q9D/K33E, we used AlphaFold2^30^ to model the three-dimensional structure of each Cbx.VD (Supplementary Fig. 6b–i). In agreement with previous studies, Cbx1, 3, and 5 are characterized by a large electronegative histone peptide-interacting surface, whereas PcG chromodomains display a more hydrophobic surface^26^. As expected, the Q9D/K33E substitutions increased the electronegative potential of the peptide-binding surface, and therefore, may establish favorable interactions with the positively charged histone peptides.

Next, we assessed specificity in greater detail by measuring binding of each wild-type chromodomain (except Cbx4) and its variant across a panel of histone peptides. To enhance detection sensitivity, we expressed each chromodomain as a fusion to the N-terminus of an engineered firefly luciferase^31^ and measured binding by luminescence intensity. In all cases, the results (Fig. 2b) were consistent with the results of the FP assays (Table 3), and binding was strongest for peptides containing trimethylated lysine residues. The chromodomains of Cbx1, Cbx3 and Cbx5 bound only to peptides that were methylated at the H3K9 position, and in the case of Cbx3, binding was significantly stronger for the engineered variant compared with the wild-type chromodomain. In contrast, the chromodomains of Cbx2, Cbx7, and Cbx8 bound to peptides that were methylated at either the H3K9 or H3K27 position, and in every case, the variants bound more strongly than the wild-type. Indeed, Cbx8.wt did not bind detectably to any peptides and Cbx2.wt did not bind detectably to peptides methylated at position H3K9, but both variants (Cbx2.VD and Cbx8.VD) exhibited robust and selective binding. Notably, in agreement with previous studies on the binding specificity of the mouse Polycomb proteins^19^, Cbx7.wt and Cbx7.VD exhibited binding to several additional peptides, but Cbx7.VD bound most strongly to the H3K9me3 and H3K27me3 peptides. Finally, Cbx6.wt did not bind to any peptides, whereas Cbx6.VD exhibited strong binding to the H3K9me3 peptide and weak binding to the H4K20 peptide, and this interaction was observed previously for Cbx6.wt^19^.

Taken together, these results showed that the Asp9/Glu33 double-substitution provides a general means for enhancing the affinities of most chromodomains of the Cbx family, without altering their specificity significantly.

### Functional characterization of high-affinity chromodomains in mESCs

**Fig. 3:**
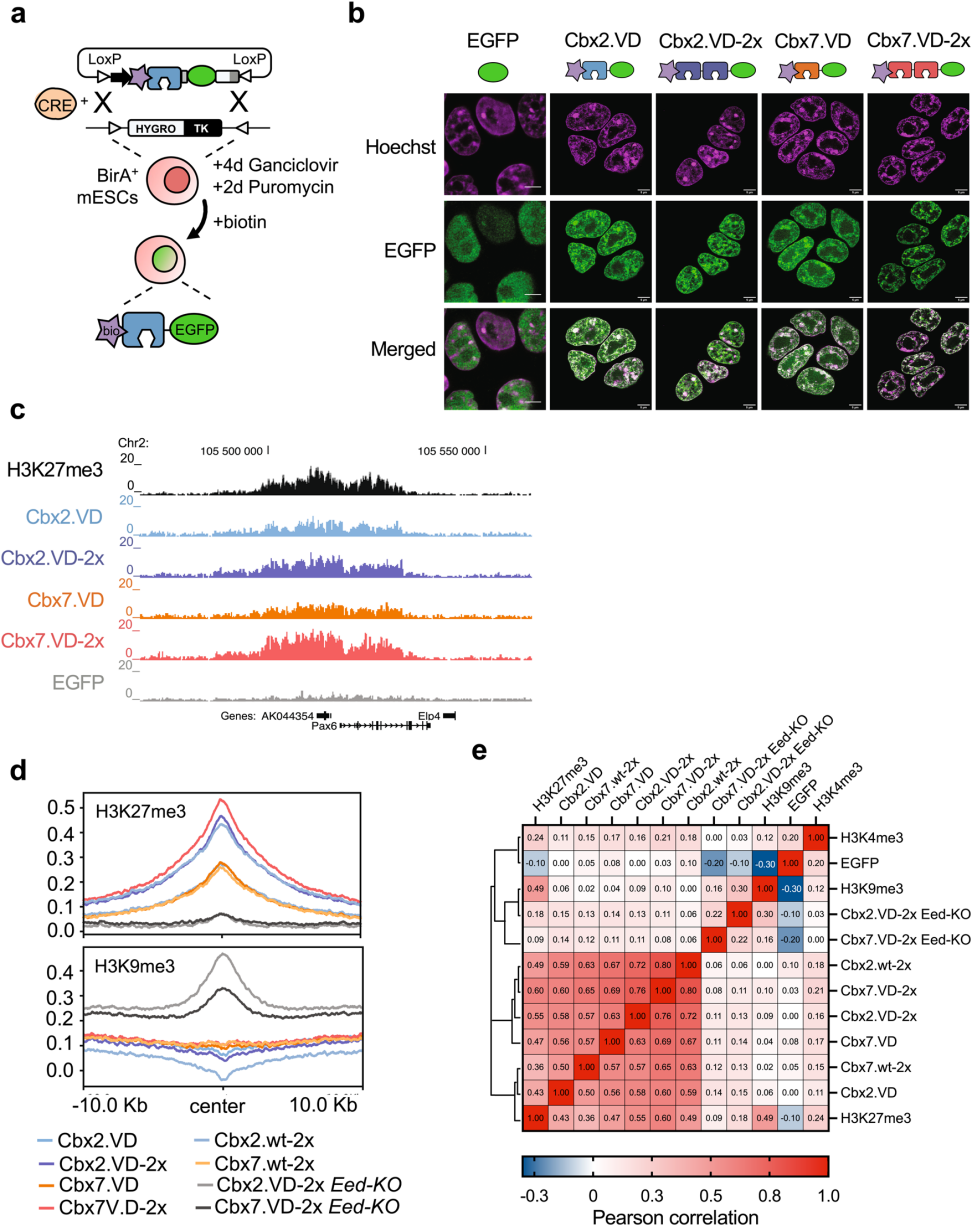
Functional characterization of engineered chromodomains in mESCs. **a** Schematic representation of RCME strategy for site-specific integration of monovalent and bivalent Cbx chromodomain variants in the genome of mESCs. Site-specific biotinylation of stably integrated chromodomains was performed in vivo by the enzymatic activity of an *E.coli* biotin ligase (BirA). **b** Live-cell imaging of mESCs expressing Cbx7.VD and Cbx2.VD chromodomains arranged in single-domain or double-domain configuration. Nuclear EGFP was used as a control. Data show representative confocal images from two independent experiments. Scale bar 5 µm. **c** Genome browser example showing correct localization of engineered chromodomains at a H3K27me3 genomic region detected by antibody ChIP–seq. Shown is the bigWig coverage-track at 100-bp intervals and normalized to Reads Per Kilobase per Million mapped reads (RPKM). Gene models are indicated. **d** The top panel shows average density profiles of EGFP-fused chromodomains centered at H3K27me3 peaks. Chromodomain variants fused to EGFP were stably integrated into mESCs or *Eed*-*KO* cells lacking H3K27me3. Data indicate increased genome-wide binding for high-affinity chromodomains, with Cbx2.VD-2x and Cbx7.VD-2x showing the greatest enrichment. The bottom panel shows the average density plots for EGFP-fused Cbx variants centered at H3K9me3 peaks. Data show lack of interaction between Cbx variants and H3K9me3-marked histones in mESCs. Both Cbx2.VD-2x and Cbx7.VD-2x displayed significant genome-wide binding to H3K9me3 peaks in mESC *Eed-KO* cells lacking H3K27me3 marks. **e** Pearson correlation analysis showing genome-wide interaction of chromodomain variants with histone modifications. Pearson correlation was obtained from comparisons of engineered chromodomains to histone modifications at filtered 1-kb-sized genomic bins (*n* = 61,145) on chromosome 19.

To explore activities of the high-affinity chromodomains in live-cells, we integrated Cbx3.wt, Cbx3.VD, Cbx5.wt, Cbx5.VD, Cbx7.VD, or Cbx2.VD into a defined site in the genome of mouse embryonic stem cells (mESCs) by recombinase-mediated cassette exchange^32^, in either a single-domain or tandem double-domain configuration (Cbx.VD-2x; Supplementary Fig. 9a). Each domain was fused to an N-terminal biotin acceptor site to allow immunoprecipitation, and at the C-terminus, to a nuclear localization signal (NLS) followed by EGFP to enable live-cell imaging (Fig. 3a, Supplementary Fig. 9a and Supplementary Dataset 4). Flow cytometry analysis of cell lines showed homogeneous and stable expression of each biotin-Cbx-EGFP fusion (Supplementary Fig. 9b). Live-cell imaging of mESCs expressing Cbx3.VD, Cbx5.VD, Cbx7.VD or Cbx2.VD showed punctate nuclear localization with accumulation at chromocenters, the nuclear periphery, and around nucleoli, in contrast to the diffuse pattern observed for EGFP alone (Fig. 3b and Supplementary Fig. 9c). Strikingly, this localization pattern of the high-affinity single-domain proteins was similar to what we previously observed for Cbx7.wt arranged in the tandem double-domain configuration (Cbx7.wt-2x), whereas the single-domain Cbx7.wt did not exhibit specific nuclear localization^16^. Lastly, cells expressing high-affinity double-domain proteins (Cbx2.VD-2x and Cbx7.VD-2x) displayed even more discrete punctate staining, with both engineered chromodomains accumulating at the nuclear periphery and around nucleoli (Fig. 3b).

We measured the genome-wide binding patterns of the high-affinity chromodomains using biotin-mediated chromatin immunoprecipitation followed by sequencing (biotin ChIP–seq)^32^. Genome-wide binding analyses revealed specific enrichment at regions marked with H3K27me3 for high-affinity single-domain and double-domain proteins (Fig. 3c, d top). Notably, Cbx2.VD and Cbx7.VD displayed similar enrichments at H3K27me3 sites compared to our previously engineered H3K27me3 chromatin reader containing double-domain Cbx7.wt-2x^16^, but both were significantly outperformed by their own double-domain versions (Cbx2.VD-2x and Cbx7.VD-2x). These results highlight the importance of multivalent interactions for efficient histone-mark recognition in cells (Fig. 3c).

These interactions were specific, as we observed genome-wide association of both Cbx2.VD-2x and Cbx7.VD-2x to the same histone modifications recognized by the wild-type chromodomains (Fig. 3e). We did not observe detectable binding signals for high-affinity single-domain or double-domain proteins at H3K9me3 sites in wild-type mESCs (Fig. 3d bottom and Supplementary Fig. 9d, e). However, when Cbx2.VD-2x or Cbx7.VD-2x were expressed in cells that lacked H3K27 methylation (*Eed-KO* cells), each domain displayed loss of binding to H3K27me3 regions but gained the ability to specifically interact with H3K9me3 sites, such as chromocenters (Fig. 3d bottom and Supplementary Fig. 9c–f). These results agreed with our previous findings^16^ and showed that, despite their ability to interact with both H3K9me3 and H3K27me3 marks in vitro, the chromodomains of Cbx2 and Cbx7 preferably recognized H3K27me3 modified histones in vivo independently of their expression levels (Supplementary Fig. 9g).

In summary, these results showed that engineered high-affinity chromodomains display strong binding to H3K27me3 marks in living cells, while maintaining the binding specificities of their wild-type counterparts.

### Effects of high-affinity chromodomains on CRISPRi efficiency

Clustered Regularly Interspaced Short Palindromic Repeats (CRISPR) interference (CRISPRi) is a powerful method for sequence-specific repression of gene expression that uses a recombinant protein consisting of a transcriptional repression domain fused to catalytically inactive Cas9 (dCas9), and a guide RNA for specific targeting^33^. The most widely used CRISPRi repressors contain a dCas9 fusion to the Krüppel-associated box (KRAB) of KOX1 (a member of the KRAB C2H2 zinc finger family) or to a bipartite repressor obtained by fusing the same KRAB domain to Methyl-CpG Binding Protein 2 (MeCP2)^34^. Although these repressors are fairly efficient, in many cases CRISPRi achieves only partial gene silencing, with repression efficiency variations depending on the cell line and the position of the targeted transcriptional start site (TSS)^34^. Thus, there is a need for more efficient repressors.

**Fig. 4:**
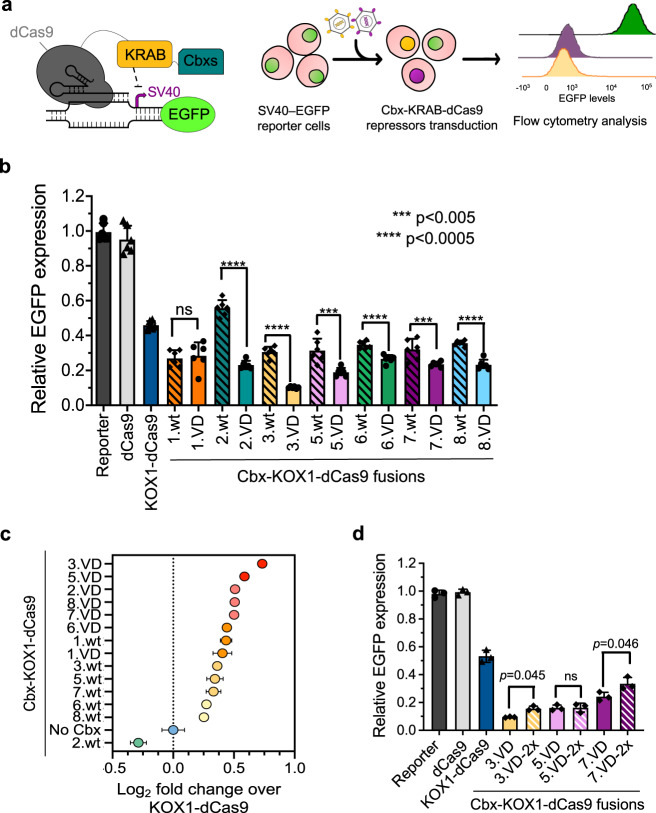
Effects of high-affinity chromodomains on CRISPRi efficiency. **a** Schematic representation of the reporter cell line and CRISPR-based repressors used in the assay. EGFP expression is driven by an SV40 promoter and Cbx-KRAB-dCas9 fusions are recruited to the SV40-EGFP reporter by stably expressing a gRNA targeting the SV40 promoter. KOX1-dCas9 and Cbx-KOX1-dCas9 repressors were delivered into the reporter cell line by lentiviral infection and EGFP silencing was measured by flow cytometry. **b** EGFP expression levels were measured by flow cytometry 20 days after cell infection. Each bar represents the geometric mean of fluorescence signals relative to untransduced reporter cells (*n* = 6, ±1 SD), with each data point showing a biological replicate. **c** Log_2_ fold change repression efficiency of Cbx-KOX1-dCas9 fusions in comparison to KOX1-dCas9 (no Cbx). Data are as in **b** and show greater EGFP repression for Cbx-KOX1-dCas9 fusions than KOX1-dCas9 (*n* = 6, *p* < 0.005, two-tailed unpaired t-test). Cbx3.VD-KOX1-dCas9 fusion showed significantly enhanced repression potency relative to KOX1-dCas9 and outperformed even the second-best repressor (Cbx5.VD-KOX1-dCas9, *n* = 6, *p* = 0.0018, two-tailed unpaired *t*-test). Some error bars are too small to be visible. **d** Comparison of the repression by KOX1-dCas9 fused to single (Cbx3.VD, Cbx5.VD, Cbx7.VD) or double (Cbx3.VD-2x, Cbx5.VD-2x, Cbx7.VD-2x) chromodomains. EGFP fluorescence signals were measured by flow cytometry 14 days after antibiotic selection of repressor-transduced cells. Data are mean of three independent experiments ± 1 SD and a two-tailed unpaired *t*-test was used to compare the reduction of EGFP levels across repressors. “ns” indicates non-significant *p* value.

Chromodomains of the PcG and HP1 families recognize repressive epigenetic marks^35,36^, and fusion of the chromodomain of Cbx5 to the KRAB domain of KOX1 enabled superior gene repression in comparison to the KRAB domain alone^34^. We reasoned that using high-affinity chromodomains in tandem with the KRAB domain of KOX1 would further enhance gene repression. To test this hypothesis, we individually fused each Cbx chromodomain (except Cbx4) and its high-affinity variant to the N-terminus of the KRAB domain of KOX1 fused to dCas9 (Supplementary Dataset 5), and we assessed their ability to silence EGFP expression in the HEK293T pSV40–EGFP reporter cell line^37^ (Fig. 4a).

Cbx-KOX1-dCas9 fusions were delivered by lentiviral infection into HEK293T cells stably expressing EGFP from a SV40 promoter, and EGFP expression was measured by flow cytometry (Fig. 4a, b and Supplementary Fig. 10). All repressors decreased EGFP expression in comparison to reporter-only cells or reporter cells transduced with dCas9 only. The KRAB domain of KOX1 reduced expression of EGFP by ~50%, whereas, almost all Cbx-KOX1-dCas9 fusions were more potent. In fact, except for Cbx2.wt, Cbx.wt-KOX1-dCas9 fusions reduced the expression of EGFP by 65% on average, with no significant differences between the domains. Notably, Cbx.VD-KOX1-dCas9 fusions containing high-affinity chromodomains enabled stronger repression of EGFP expression, and remarkably, the fusion containing Cbx3.VD reduced EGFP levels by ~90%.

The extent of repression correlated strongly with the affinity of the domains for H3K9me3 and H3K27me3 marks. For example, while we did not observe a marked difference in repression by Cbx1.wt compared with Cbx1.VD, in agreement with their nearly identical affinities for H3K9me3 peptides (Table 3), Cbx2.VD provided 76% repression, whereas the lower affinity Cbx2.wt reduced EGFP expression by only 44%. Repression differences were not dependent on the expression levels of the different repressors, as western blot analysis showed that even poorly expressed repressors could strongly reduce EGFP levels (Supplementary Fig. 11a). We compared the repression efficiency of each chromodomain fusion to that of KOX1-dCas9 and found that the high-affinity chromodomain fusions showed on average a 40% improvement in repression (Fig. 4c). Most impressively, the Cbx3.VD fusion displayed 66% greater repression than KOX1-dCas9 and was 10% better than the second-best repressor containing the Cbx5.VD fusion.

To further evaluate the correlation between the affinity of chromodomains and their repressive activity, we delivered by lentiviral infection double-domain versions of Cbx-KOX1-dCas9 fusions containing two copies of Cbx3.VD, Cbx5.VD, or Cbx7.VD arranged in tandem and compared their repressive potential to single-domain high-affinity chromodomain fusions (Fig. 4d). As previously observed, fusion of chromodomains to KOX1-dCas9 significantly reduced the expression levels of EGFP, but in general, each double-domain fusion performed worse than its single-domain counterpart. Cbx3.VD-KOX1-dCas9 was the strongest repressor, and it was highly potent even when transiently transfected into HEK293T SV40-EGFP reporter cells (Supplementary Fig. 11b).

**Fig. 5:**
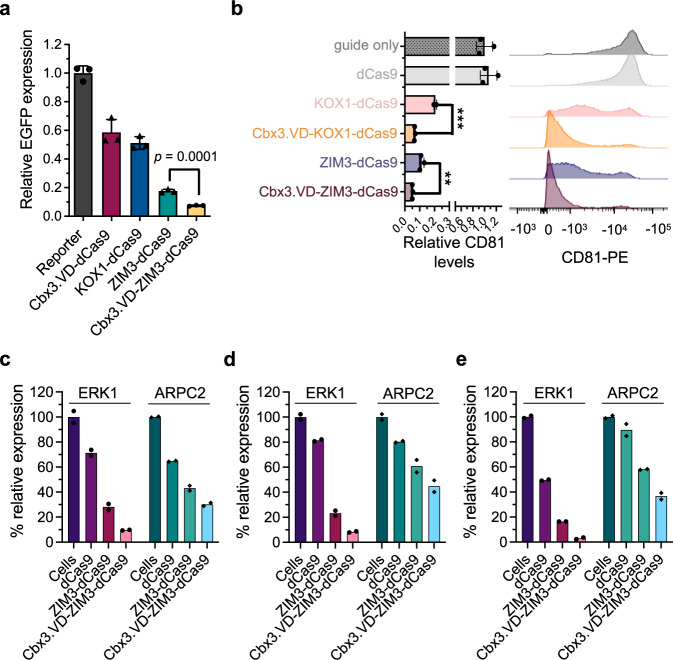
Cbx3.VD enables modular and potent gene repression. **a** Relative EGFP levels of HEK293T-SV40 EGFP reporter cells stably transduced with different KRAB domain-containing repressors (KOX1-dCas9, ZIM3-dCas9, and Cbx3.VD-ZIM3-dCas9) and a repressor lacking KRAB domains, obtained by directly fusing Cbx3.VD to dCas9 (Cbx3.VD-dCas9). Histograms show the mean from three independent experiments ±1 SD of EGFP levels relative to untransduced reporter cells. **b** Repression of endogenous *CD81*. CRISPRi repressors were recruited to *CD81* TSS and CD81 surface expression levels were measured by flow cytometry 4 days after antibiotic selection of repressor-transduced cells. Left panel shows CD81 surface levels relative to HEK293T cells transduced only with gRNA targeting *CD81* promoter (guide only). Data are mean from three independent measurements ± 1 SD, asterisks indicate *p* values (two-tailed unpaired *t*-test, **p = 0.002, ***p = 0.0001). Some error bars are too small to be visible. Right panel shows representative flow cytometry profiles of CD81 expression detected with an anti-CD81 PE-conjugated antibody. **c**–**e** Comparison of the repressive potency of Cbx3.VD-ZIM3-dCas9 with that of ZIM3-dCas9 and dCas9. CRISPRi repressors were recruited to *ERK1* and *APRC2* loci and their protein expression levels in **c** HEK293T, **d** U20S, and **e** HeLa cells were measured by western blot. The percentage relative expression was determined by densitometry and was normalized to the band intensity of untransduced cells (mean ± 1 SD; from two biological replicates). See Supplementary Fig. 12 for representative western blots.

To evaluate the generality of our method for developing potent CRISPR-based repressors, we combined Cbx3.VD with the KRAB domain of ZIM3, which showed the strongest repressive potency among currently used CRISPRi repressors^38^ (Fig. 5a). A Cbx3.VD-dCas9 fusion (lacking the KRAB domain) reduced EGFP expression to levels comparable to the KOX1-dCas9 fusion, whereas a fusion of dCas9 to the ZIM3 KRAB domain (ZIM3-dCas9) had a stronger repressive effect on EGFP levels. Most notably, fusion of both Cbx3.VD and the ZIM3 KRAB domain to dCas9 (Cbx3.VD-ZIM3-dCas9) further improved repressive potency and reduced EGFP levels by 92%.

Finally, we assessed whether Cbx3.VD-KRAB domain fusions could outperform current versions of CRISPRi for repression of endogenous genes. We recruited repressors to the promoter of *CD81* by lentiviral infection of HEK293T cells and assayed gene silencing by flow cytometry. CRISPRi repressors significantly silenced *CD81* expression, and strikingly, the additional fusion of Cbx3.VD to either the KRAB domain of KOX1 or ZIM3 greatly enhanced repression and nearly abolished cell surface levels of the CD81 protein (Fig. 5b). We further validated the robustness of the Cbx3.VD-ZIM3-dCas9 repressor by targeting two additional genes (*ERK1* and *ARPC2*) in three different cell lines (HEK293T, HeLa and U20S) (Fig. 5c–e and Supplementary Fig. 12). Cbx3.VD-ZIM3-dCas9 displayed efficient repression of targeted genes in each of the tested cell lines, and improved gene silencing, relative to ZIM3-dCas9, in every case except for ARPC2 in U20S cells (Fig. 5d).

Taken together, these results showed that engineered high-affinity chromodomains can be used to enhance gene repression in CRISPRi applications beyond what is possible with the best methods available currently.

## Discussion

Here we have presented a modular strategy for enhancing the affinity of chromodomains of the PcG and HP1 families without affecting their specificity in vitro or in vivo. We used a phage-displayed library to identify key substitutions that greatly improved the binding of chromodomains to H3K9me3 and H3K27me3 peptides. While we and other groups have previously engineered HP1 chromodomains naturally characterized by sub-micromolar affinity^22–24^, in this study we focused on the weakly interacting chromodomain of Cbx7 to enhance its affinity for H3K27me3. We used phage-displayed libraries to isolate Cbx7 variants with higher affinity for H3K27me3 peptides and identified a variant (Cbx7.VD) that improved affinity by >20-fold. Mutational analysis revealed that only two substitutions were required for affinity enhancement, and genome-wide binding experiments in cells showed that these substitutions did not affect specificity. Neither Cbx2.VD nor Cbx7.VD bound to H3K9me3 sites in mESCs when expressed at levels comparable to endogenous Cbxs, suggesting that both chromodomains preferably recognize H3K27me3 sites in vivo, despite their ability to interact with both marks in vitro. However, the current data cannot exclude that chromodomain concentrations far exceeding the available H3K27me3 sites in cells might result in binding to H3K9me3 marks in vivo.

Introduction of the two affinity-enhancing substitutions into all eight Cbx chromodomain family members resulted in enhanced affinity in 6 out of 8 chromodomains, as the affinity of Cbx1 was not altered, whereas the substitutions were detrimental in Cbx4. These results demonstrated the modularity of our approach and allowed us to develop a general method to significantly enhance the affinity of most chromodomains of both PcG and HP1 family members without affecting their specificity.

Methylation of H3K27 is critical for establishing and maintaining cell-specific transcriptional programs, yet we are just beginning to understand how PcG variant complexes are recruited to chromatin^39^. Therefore, the identified substitutions that enhance the affinity of Cbx chromodomains for H3K27me3 marks will prove helpful in uncovering the physiological role of endogenous Cbx proteins that are found in PRC1 variant complexes.

Several studies have taken advantage of recombinant epigenetic reader domains as molecular tools to investigate chromatin biology^11,12,22^. However, our data showed that the weak affinity of natural epigenetic readers is insufficient for effective binding to histone modifications and that enhancing the affinity of chromodomains ensures deeper genome coverage under physiological conditions. Additionally, we showed that combining enhanced affinity with multivalent interactions ensures efficient nuclear localization and detection of chromatin modifications in vivo. Furthermore, the intrinsic specificities and weak affinities of natural epigenetic reader domains might result in binding to multiple modified histones and lower chromatin recovery than with antibodies in ChIP-seq experiments^40^, thereby highlighting the need for engineered domains with desired binding specificities and enhanced affinities.

The CRISPR-Cas9 system has emerged as a powerful tool for genome engineering, but Cas9 endonuclease activity can induce cellular toxicity as well as irreversible modifications at cut sites, limiting its applications^41^. Therefore, regulation of gene expression using catalytically inactive dCas9 presents an attractive alternative^33^. Previous work has shown that fusion of dCas9 to transcriptional regulators enables the development of transcriptional repressors^42^, albeit with limited efficacy^43^. Nanobody-mediated recruitment of endogenous full-length HP1 family members (Cbx1, Cbx3, and Cbx5) to chromatin allowed potent gene silencing, but repression was dependent on affinity and the availability of target-specific nanobodies^44^.

Consequently, to provide a modular strategy for the development of potent repressors, we combined the intrinsic transcriptional repressive activity of human Cbx proteins with repressive KRAB domains fused to dCas9. Fusion of Cbx chromodomains to the KRAB domain of KOX1 and dCas9 enabled cooperative increase in repressive activity, as KRAB domains recruit the KAP1 co-repressor, SETDB1, HP1, and the NuRD histone deacetylase complex, resulting in higher H3K9me3 and H3K27me3 levels^45^.

We showed that the repressive potency of Cbx-KRAB-dCas9 fusions positively correlated with the affinity of the chromodomains. However, further affinity enhancement obtained with bivalent chromodomains resulted in reduced repressive activity. This effect is likely due to the sequestration of the Cbx-KOX1-dCas9 repressors at trimethylated histone sites and consequent interference with the gRNA:dCas9 interaction, as samples treated with a broad-spectrum methyltransferase inhibitor displayed further weakened repression (Supplementary Fig. 13).

We identified Cbx3.VD-KOX1-dCas9 as a highly potent transcriptional repressor and demonstrated that fusion of Cbx3.VD to another KRAB domain from ZIM3 allowed the development of transcriptional repressors with superior potency to current gold standards. Overall, our results showed that high-affinity chromodomains are powerful tools for genome-wide binding analysis, live-cell imaging, and dCas9-based gene repression, and thus, they represent a powerful platform to dissect chromatin biology and to enhance gene silencing.

## Methods

### Synthetic peptides

All peptides were synthesized by GenScript, desalted and verified by mass spectrometry. Unless otherwise noted, peptides were modified at the N-terminus with biotin followed by an aminohexanoic acid linker. For fluorescence polarization experiments, H3K9me3 and H3K27me3 peptides, as well as H3K9 and H3K27 non-methylated peptides, were labeled at the N-terminus with fluorescein isothiocyanate (FITC). See Supplementary Table 1 for peptide sequences.

### Construction of Cbx7-chromo library

The phage-displayed Cbx7.V library was constructed by site-directed mutagenesis^46^ of a phagemid designed for the display of Cbx7-chromo on the major coat protein P3 of the M13 bacteriophage. A total of 16 residues encompassing two regions involved in methyllysine recognition (region 1: residues 7–12; region 2: positions 32–41) were diversified simultaneously with a soft randomization strategy^29^. Mutagenic oligonucleotides, which sequence is reported in Supplementary Table 3, were synthesized to adjust the nucleotide ratio of diversified positions to 70% of the wild-type nucleotide and 10% of each of the other nucleotides. The diversity of the obtained Cbx7.V library was 1.1 × 10^10^.

### Selection of Cbx7 variants

For selection of Cbx7 variants (Cbx7.Vs), streptavidin was immobilized on 96-well Nunc-Immuno MAXISORP plates (Thermo Scientific) at 2 µg/ml in PBS pH 7.4, overnight at 4 °C. Wells were blocked by addition of 300 µl/well of PBS pH 7.4, 0.5% BSA (PB buffer) and incubation at 25 °C for 1 h. Biotinylated H3K27me3 or H3K27 peptides were added to wells at 2 µg/ml in PBS pH 7.4 and incubated at 25 °C for 30 min. Unbound peptide was removed by washing wells three times with 300 µl/well of PBS pH 7.4, 0.05% Tween-20 (PT buffer). The phage-displayed library was cycled through five rounds of binding selections with the immobilized biotinylated H3K27me3 peptide as previously described^47^. To remove form the phage pool clones that bound to non-methylated targets, the Cbx7-chromo library was pre-incubated for 1 h at 25 °C on plates with an immobilized, biotinylated H3K27 peptide prior to transfer to plates containing the H3K27me3 peptide.

Phage ELISAs were performed to identify positive clones able to bind to the H3K27me3 peptide but not to streptavidin or the unmethylated H3K27 peptide, as described^48^. Amino acid sequences of selected Cbx7.Vs were determined by Sanger DNA sequencing.

### Plasmids for bacterial and mammalian expression of chromodomains

For BLI and fluorescence polarization experiments, wild-type Cbx chromodomains, Cbx7.V1, Cbx7.V1 back-mutants and Cbx7.VDs gene fragments were amplified by PCR and cloned in frame with a His_6_ tag and Flag tag into pET21b (EMD Biosciences) using the HiFi DNA Assembly Master Mix (New England Biolabs). See Supplementary Datasets 2 and 3 for chromodomain boundaries and sequences used in this study.

For assaying in vitro binding specificity of wild-type chromodomains and Cbx.VDs, the coding sequence of the engineered YY5 firefly luciferase from *Photinus pyralis*^31^ was synthesized as a gene fragment (Integrated DNA Technologies), amplified by PCR and fused by Gibson assembly to the C-terminus of chromodomains previously cloned into the pET21b vector.

For site-specific recombination into mESCs, coding sequences of Cbx3.wt, Cbx3.VD, Cbx5.wt, Cbx5.VD, Cbx2.VD, Cbx7.VD and their bivalent versions (Cbx2.VD-2x, Cbx7.VD-2x) were synthesized as gene fragments (Integrated DNA Technologies) and cloned into the recombinase-mediated cassette exchange (RCME) targeting vector parbit-v6^16^ by Gibson assembly. The obtained Cbx.VD encoding genes contained a N-terminal acceptor peptide for site-specific biotinylation and were fused in frame with a cassette carrying a nuclear localization signal (NLS) followed by EGFP, an internal ribosome entry site (IRES) and the puromycin-*N*-acetyltransferase gene.

### Chromodomain expression and purification

*E. Coli* BL21(DE3) cells co-transformed with a plasmid for expression of a chromodomain and a plasmid for the expression of the Erv1p and DsbC chaperones^49^ were grown in 2YT medium containing 100 μg/ml carbenicillin and 34 µg/ml chloramphenicol at 37 °C with 200 rpm shaking to OD_600_ 0.4. Expression of Erv1p and DsbC was induced with 0.5% (w/vol) arabinose, and the temperature was lowered to 30 °C for 45 min, as described^49^. Expression of chromodomains was induced with 100 μM IPTG at 30 °C for 4 h. Cultures were pelleted and resuspended in 10 ml Lysis Buffer (50 mM Tris-HCl pH 7.8, 1 mM MgCl_2_, 1.0 % TritonX-100, 1 mM 1,4-Dithiothreitol (DTT), 1 mg/ml lysozyme (Bioshop), 50 U/ml Benzonase (Roche), 1 mM Phenylmethylsulfonyl fluoride (PMSF), and protease inhibitor cocktail (Sigma-Aldrich)) and incubated at 25 °C with gentle agitation for 20 min. Following incubation at 25 °C, NaCl was added to the lysate to a final concentration of 300 mM, and the lysate was subjected to two freeze and thaw cycles followed by sonication.

Protein purification was performed by standard methods with Ni-NTA resin (Qiagen). Following protein binding, Ni-NTA resin was washed twice with 30 ml of a high salt wash buffer (50 mM Tris-HCl pH 7.8, 1 M NaCl, 1 mM DTT) to remove DNA bound to chromodomains. Proteins were eluted with a 50–300 mM imidazole buffer gradient. The purity of eluted fractions was determined by SDS-PAGE, and eluted proteins were exchanged into PBS pH 7.4 containing 1 mM DTT (Sigma-Aldrich) by dialysis at 4 °C.

After elution of chromodomain-luciferase fusions, the buffer was exchanged to TBS pH 8.5 (50 mM Tris-HCl pH 8.5, 150 mM NaCl, 1 mM DTT).

For fluorescence polarization (FP) and isothermal titration calorimetry (ITC) experiments, following chromodomains expression and purification, both His_6_ and Flag tags were removed by overnight incubation at 4 °C with Tobacco Etch Virus (TEV) protease at a 1:100 protease:substrate molar ratio. After overnight incubation, TEV protease and un-cleaved Cbx proteins were removed by applying the reaction mixture to Ni-NTA resin. Unbound fractions were collected, concentrated using a 3 kDa cut-off Amicon Ultra‐4 concentrator (EMD Millipore) and flash frozen to −80 °C.

Protein concentrations were determined from OD_280_ measurements and calculated using extinction coefficients from ExPASy ProtParam^50^.

### Phage ELISA

Phage ELISA experiments were performed by coating 384-well MaxiSorp plates (Thermo Fisher) with 25 µl/well of 2 µg/ml streptavidin (New England Biolabs, N7021S) in PBS pH 7.4, and incubating them at 4 °C for 16 h. Each well was blocked with 60 µl/well of PBS pH 7.4 containing 0.5% BSA (PB buffer) and incubated at 25 °C for 1 h. Plates were washed four times with 90 µl/well of PBS pH 7.4 containing 0.05% Tween-20 (PT buffer) and incubated with 25 µl/well of biotinylated histone peptides at 2 µg/ml in PBS pH 7.4 for 30 min at 25 °C. Plates were incubated with phage stocks previously diluted three-fold in PB buffer containing 0.05% Tween-20 (PBT buffer) at 25 °C for 1 h with gentle agitation. Plates were washed and bound phage was detected using 25 µl/well of an anti–M13-HRP-conjugated antibody (1:3,000 dilution in PT buffer; SinoBiological, 11973-MM05T-H). Following incubation at 25 °C for 30 min with gentle shaking, plates were washed, and binding was assessed by adding 25 µl/well of the 3,3′,5,5′-tetramethylbenzidine (TMB) (Thermo Fisher) chromogenic substrate. Plates were incubated with TMB at 25 °C with gentle shaking for 5–10 min, and the colorimetric reaction was stopped by addition of 25 µl/well of a 1 M H_3_PO_4_ solution. Plates were read spectrophotometrically at 450 nm using a PowerWave XS microplate reader (BioTek).

### Luciferase-based assays

For luciferase-based assays, 384-well white plates (PerkinElmer) were coated for 16 h at 4 °C with 4 µg/ml streptavidin (New England Biolabs, N7021S) in TBS pH 8.5 (50 mM Tris-HCl pH 8.5, 150 mM NaCl). Each well was blocked with 60 µl/well of TBS pH 8.5 containing 0.5% BSA (TB buffer) and incubated at 25 °C for 1 h. Plates were washed four times with 90 µl/well of PBS pH 7.4, 0.05% Tween-20 (PT buffer) and incubated with 25 µl/well of biotinylated histone peptides at 5 µg/ml in TB buffer pH 8.5 for 30 min at 25 °C. Plates were washed and incubated with Cbx chromodomain-luciferase fusions diluted to 0.1 nM for Cbx.VD or 100 nM for Cbx.wt in TB buffer, 0.05% Tween-20, 1 mM DTT, 5% glycerol (TBT buffer). Plates were incubated for 1 h at 25 °C with gentle shaking, washed six times and luminescence intensity was measured with a Biotek Synergy 5 plate reader (Biotek) using the ONE-Glo^TM^ EX luciferase assay system reagent (Promega). Following background subtraction of the luminescence signal derived from wells coated with streptavidin, the luminescence intensity was normalized to wells with immobilized H3K9me3 peptide. Since no binding was detected for Cbx2.wt with immobilized H3K9me3 peptide, the luminescence intensity measured for Cbx2.wt and Cbx2.VD were normalized against wells immobilized with the H3K27me3 peptide.

### Bio-Layer Interferometry

BLI experiments were performed on an Octet HTX system (ForteBio) using biosensor tips coated with streptavidin to immobilize biotinylated H3K9me3 and H3K27me3 peptides. Binding analysis was performed by analyzing the association of biosensor-immobilized methylated peptides to Cbx7.V1 variants in solution at 5 or 1 µM. Binding assays were performed at 25 °C in BLI reaction buffer (25 mM HEPES pH 7.0, 150 mM NaCl, 1 mg/ml BSA, 0.01% Tween-20). Dissociation constants (*K*_D_) were obtained by fitting the response wavelength shifts in the steady-state regions with the Langmuir binding model.

### Fluorescence polarization binding assays

Fluorescence polarization (FP) assays were performed in a 20 µl volume at a constant histone peptide concentration of 0.5 nM. Binding measurements were performed in FP buffer (25 mM HEPES pH 7.5, 150 mM NaCl, 1 mM DTT, 0.01% TritonX-100) by mixing in a 384‐well black plate (Corning) 0.5 nM FITC‐labeled histone peptides with serial dilutions of wild-type Cbx chromodomains ranging from 200–0.2 µM. For affinity determination of Cbx7.V1 and all Cbx.VDs, chromodomains were serially diluted from 50 µM to 50 nM. Samples were incubated for 30 min at 25 °C prior to data acquisition. Fluorescence polarization was measured using an excitation filter of 485 nm and an emission filter of 530 nm with a Synergy Neo2 Multi-Mode Assay Microplate Reader (Biotek). Dissociation constants were determined using Prism v.9.3 (GraphPad Software Inc) with a one‐site total binding model.

### Isothermal Titration calorimetry

ITC experiments were performed on a MicroCal PEAQ-ITC Automated (Malvern Instruments) by titrating H3K9me3 and H3K27me3 peptides (between 1.2 and 2 mM from the syringe, depending on the experiment) into Cbx7.V1 (between 70 and 112 µM in the cell, depending on the experiment) at 25 °C in PBS pH 7.4 containing 1 mM TCEP ((tris(2-carboxyethyl)phosphine)). Dissociation constants were determined using the one-site binding model supplied in MicroCal PEAQ-ITC analysis software (version 1.1.0.1262) with the fitted offset control. Data are the average from three independent experiments ± 1 SD.

### Thermal stability measurements

For measuring thermal stability, 40 µM Cbx4.wt or Cbx4.VD protein sample in PBS pH 7.4 in a final volume of 30 μL was incubated at 25, 40, 50, 70, or 90 °C for 3 min and then cooled to 10 °C at 3 °C/s in a Bio-Rad C1000 Thermal Cycler. Samples were then spun at 17,000×*g* at 4 °C for 30 min to remove aggregates, and the supernatant was analyzed by SDS-PAGE. Gels were stained with Coomassie blue and band intensities were quantified by densitometry using a Gel Doc XR imager and Image Lab 3.0 software (Bio-Rad). Percent soluble chromodomain was calculated as ×100 (band intensity at the indicated temperature/ band intensity of samples heated at 25 °C).

### Cell culture

HEK293T (ATCC), HeLa (ATCC), and HEK293T SV40-EGFP reporter cells (a gift from the Taipale laboratory, University of Toronto) were grown in Dulbecco’s Modified Eagle Medium (DMEM) (Life Technologies) containing 10% (vol/vol) fetal bovine serum (FBS, Sigma-Aldrich) at 37 °C with 5% CO_2_, whereas U20S cells (ATCC) were grown at 37 °C with 5% CO_2_ in McCoy’s 5a Medium Modified (ATCC) containing 10% FBS. Mouse embryonic stem cells (mESCs) (HA36CB1, 129-C57Bl/6, ATCC) were grown on feeder cells or 0.2% gelatine coated dishes, in DMEM supplemented with 15% fetal calf serum (Invitrogen), 1× nonessential amino acids (Invitrogen), 1 mM L-glutamine, LIF, and 0.001% β-mercaptoethanol. Mouse embryonic stem cells were stably integrated with Cbx.VD-encoding genes as previously described^16^. Briefly, Cbx.VDs cloned into the recombinase-mediated cassette exchange (RCME) targeting vector parbit-v6^16^ were co-transfected with a Cre recombinase-encoding plasmid (1:0.6 plasmid DNA ratio) into RCME competent mESCs that stably expressed *E.coli* biotin ligase (BirA) for site-specific biotinylation of Cbx.VDs. Transfected cells were selected for 4 days with 3 mM ganciclovir, followed by a second selection round with 2 mM puromycin for 2 days. The *Eed*-*KO* cell line was generated and cultured as previously described^16^. All cell lines were routinely tested for mycoplasma contamination.

### Plasmids for lentivirus production

Genes encoding Cbx-KRAB domain fusions were synthesized as gene fragments (Integrated DNA Technologies) and cloned with the Gateway BP Clonase II system (Thermo Fisher, 11789020) into the Gateway Entry vector pDONR221 (Thermo Fisher) according to the manufacturer’s protocols. Cbx-KRAB domain fusions cloned into pDONR221 were then transferred, via Gateway LR Clonase II system (Thermo Fisher, 11791020), into the pLX303-dCas9 vector^38^ (a kind gift of Dr. Taipale, University of Toronto). The pLX303-dCas9 vector encoded a *Streptococcus pyogenes* dCas9 with two C-terminal and one N-terminal SV40 nuclear localization signals. See Supplementary Dataset 5 for sequences of all dCas9-repressors used in this study.

### Lentivirus production and generation of stable cell lines

Lentivirus was produced by transiently transfecting low passaged HEK293T cells seeded in 6-well plates, 2 ml/well, at 0.3 × 10^6^ cells/ml. Cells were transfected in Opti-MEM (Thermo Fisher) with 1.25 µg of each chromodomain-encoding plasmid, 900 ng of the lentiviral packaging plasmid psPAX2 and 150 ng of envelope plasmid pVSV-G using Lipofectamine 2000 (Thermo Fisher) according to the manufacturer’s instructions. Six hours post transfection, media was replaced with DMEM containing 10% FBS and 11% BSA. Viral particles were harvested 48–72 h post transfection and filtered through a 0.45 µm filter.

For generation of cells stably expressing Cbx-KRAB-dCas9 fusions, HEK293T, HeLa, U20S and HEK293T SV40-EGFP reporter cells were seeded in six-well plates at 0.3 × 10^6^ cells/ml and transduced overnight in complete media containing 10% FBS and 8 µg/ml hexadimethrine bromide (Polybrene, Sigma-Aldrich) at high multiplicity of infection with viral particles containing each repressor-encoding plasmid. Following overnight incubation, lentiviral particles were removed, and transduced cells were selected with complete media containing 10% FBS and blasticidin. For selection of stably transfected HEK293T and HEK293T SV40-EGFP, cells were treated with 6 µg/ml blasticidin for 6 days. HeLa cells transduced with Cbx-KRAB-dCas9 fusions were selected by culturing cells for 4 days in DMEM containing 10% FBS and 4 µg/ml blasticidin. Transduced U20S cells were selected by culturing cells in McCoy’s 5a Medium Modified containing 10% FBS and 5 µg/ml blasticidin for 4 days.

For *CD81, APRC2* and *ERK* repression experiments, lentivirus containing a plasmid encoding one guide RNA (gRNA) targeting *CD81*, two gRNA-encoding plasmids for *APRC2* and *ERK* were produced by transiently transfecting low passaged HEK293T cells with 900 ng of the psPAX2 plasmid, 150 ng of the pVSV-G vector and 1.25 µg of the pLCKO vector expressing the U6-driven *CD81, APRC2* and *ERK* gRNAs (see Supplementary Dataset 6 for gRNA sequences)^38^ (a kind gift from Mikko Taipale laboratory, University of Toronto). Transfections were performed using Lipofectamine 2000 (Thermo Fisher) according to the manufacturer’s instructions. Viral particles were harvested 72 h post transfection, filtered through a 0.45 µm filter and used to infect overnigh HEK293T, HeLa and U20S cells previously transduced with CRISPRi repressors. Following overnight incubation, lentiviral particles were removed, and transduced cells were selected using complete media containing puromycin. Transduced HEK293T and HeLa cells were grown for 3 days in DMEM containing 10% FBS and 2 µg/ml or 6 µg/ml puromycin, respectively. To isolate transduced U20S, cells were grown in McCoy’s 5a Medium containing 10% FBS and 5 µg/ml puromycin for 4 days. HEK293T, HeLa and U20S cells stably expressing both gRNAs and CRISPRi repressors were maintained for 4 days before flow cytometry and western blot analysis.

### Time course assessment of EGFP repression

HEK293T-SV40 EGFP cells were seeded into glass bottom Incucyte® Imagelock 96-well plates (Essen Bioscience) at 0.4 × 10^5^ cells (100 µl/well). Cells were transfected with 0.3 µg plasmids encoding Cbx3.VD-KOX1-dCas9, KOX1-dCas9 and dCas9 only using Lipofectamine 2000 (Thermo Fisher) according to the manufacturer’s instructions. Twelve hours post transfection, media was replaced, and plates were incubated for 168 h in the Incucyte® live-cell analysis system (Essen Bioscience) with automatic image acquisition every 3 h.

### Flow cytometry

To determine expression levels of Cbx-EGFP fusions integrated into mESC genome, cells were resuspended in DPBS and stained with LIVE/DEAD Fixable Near-IR Dead Cell Stain (Invitrogen, L34975) to assess cell viability. Cells were analyzed on a FACSCanto flow cytometer (BD Biosciences) using the EGFP (Alexa Fluor 488-A), and live/dead (APC-Cy7A) filters to detect live cells expressing EGFP.

To assess the repressive potential of each Cbx-KRAB-dCas9 fusion, stably transfected HEK293T SV40-EGFP reporter cells were harvested by trypsinization, washed once with ice-cold DPBS, and resuspended in PBS containing 1% BSA and 0.1% NaN_3_ (FACS-A buffer) at 2.5 × 10^6^ cells/ml.

To determine the correlation between methylation and chromodomain-mediated enhanced gene repression, cells were treated for 24 h with 10 µM chaetocin (StemCell technologies) dissolved in DMSO prior to flow cytometry analysis. As a control, cells were incubated for 24 h with DMSO in absence of the histone lysine methyltransferase inhibitor.

For *CD81* repression analysis, HEK293T cells stably expressing *CD81* gRNA and CRISPRi repressors were harvested by trypsinization, washed once with ice-cold DPBS, and resuspended in in FACS-A buffer at 2.5 × 10^6^ cells/ml. Labelling of cell-surface CD81 was performed by incubating 2.5 × 10^5^ cells with 25 µl of the phycoerythrin (PE)-conjugated mouse anti human CD81 antibody (clone JS-81, BD Biosciences) on ice for 30 min. Antibody excess was removed by centrifugation at 390×*g*, 4 °C for 3 min. Cells were washed three times with FACS-A buffer and then resuspended in the same buffer at 2.5 × 10^6^ cells/ml. Cells were analyzed on a LSR Fortessa X20 flow cytometer (Becton Dickinson), and data were analyzed with FlowJo Software (version 10.1, FlowJo, LLC).

### Live-cell imaging

Live-cell imaging experiments were performed by seeding mESCs expressing Cbx.VDs (0.2 × 10^5^ cells) on a 0.2% gelatin-coated 35-mm chambered coverslip (Ibidi, 80826) 1 day before imaging. Nuclei were stained with Hoechst 33342 (Invitrogen, 62249) for 10 min, washed twice with DPBS and covered with embryonic stem cell medium containing DMEM without phenol red (Invitrogen, 31053028). Images were acquired with a P5 inverted confocal laser scanning microscope (Leica) equipped with a climate chamber and an argon laser, using the GFP (excitation BP 470/40, emission BP 525/50) and N3 (excitation BP 546/12, emission BP 600/400) filters. Image analysis was performed using Fiji software (version 2.0.0)^51^. Appropriate single z planes were selected for image display.

### Chromatin immunoprecipitation sequencing

ChIP samples were collected from two biological replicates (independent mESC cell lines). For chromatin extraction, 30–50 × 10^6^ cells were trypsinized, washed once in PBS and fixed with 1% formaldehyde for 8 min at 25 °C, followed by reaction quenching with addition of glycine to a final concentration 0.12 M and incubation on ice for 10 min. Cells were washed twice in 10 ml ice-cold PBS, followed by centrifugation at 680×*g* for 5 min. Cells were resuspended and incubated for 10 min in 10 ml Paro Rinse 1 buffer (10 mM Tris pH 8.0, 10 mM EDTA, 0.5 mM EGTA) on ice, followed by centrifugation at 680×*g* for 5 min. Cells were resuspended in 10 ml of Paro Rinse 2 buffer (10 mM Tris pH 8.0, 0.25% Triton X-100, 1 mM EDTA, 0.5 mM EGTA and 200 mM NaCl), filtered for three times through a prechilled G26 syringe, and incubated for 10 min on ice followed by centrifugation at 680×*g* for 5 min. Final cell lysis was performed with Mnase Digest buffer (20 mM Tris pH 8.0, 5 mM MgCl_2_, 1 mM CaCl_2_, 10 mM NaCl, 0.25 M Sucrose, 1% Triton X-100, supplemented with protease inhibitor cocktail mix (Roche, 11836170001)) in a concentration-dependent volume (16,500 cells per microliter) for 0.5 to 1 h on ice. Cross-linked chromatin was subjected to digestion with MNase (New England Biolabs) by adding 50 units of MNase per 1 ml of buffer and incubating the reaction at 37 °C for 20 min with repeated stirring. The MNase digestion was stopped by adding 10x Stop buffer to a final concentration of 150 mM NaCl, 100 mM EDTA, 200 mM EGTA on ice and SDS to 0.1 % final concentration. MNase-digested chromatin was subjected to sonication (four cycles of 32”ON/50”OFF) in a Bioruptor Pico instrument (Diagenode) according to the manufacturer’s instructions. Sonicated chromatin was centrifuged at 12,000×*g* for 10 min at 4 °C and supernatant was used for further steps. For biotin ChIP-seq experiments, 100 µg chromatin extracted from mESCs expressing biotinylated Cbx.VDs were applied to 30 µl of streptavidin-coated M280 magnetic beads (ThermoFisher) previously blocked for 1 h at 4 °C with 1% cold fish skin gelatin (Sigma-Aldrich, G7041) and 100 ng yeast tRNA (Thermo Fischer, 15401011) supplemented with protease inhibitor cocktail mix (Roche). Chromatin was incubated with beads at 4 °C, overnight with end-over-end rotation. Following overnight incubations, beads were washed and chromatin was eluted as previously reported^52^. ChIP-seq libraries were prepared using the NEBNext Ultra II DNA library prep Kit for Illumina (New England Biolabs, E7645L) according to the manufacturer’s instructions. Samples tagged with unique index barcodes were combined at equal molar ratios and sequenced as pools on a NovaSeq 6000 sequencing system (Illumina) according to Illumina standards with 100 base-pair single-end sequencing. Library demultiplexing was performed following Illumina standards.

### Genomic data analysis

Adaptor sequences were removed using Trim Galore (https://github.com/FelixKrueger/TrimGalore). Trimmed reads were mapped to the mm9 mouse genome reference using Bowtie2 version 2.3.5.1^53^ using the --no-unal and --very-sensitive options. Alignments were stored as BAM files, low-quality reads (excluding reads with mapping quality <40) were filtered out using SAMtools 1.11^54^, and identical reads from PCR duplicates removed with Picard Toolkit (version:2.23.9, https://broadinstitute.github.io/picard/). BAM files were then converted to bigWig files for visual inspection in genome browsers, and GC-bias was checked and corrected using the correctGCBias module of deepTools 3.5.0^55^. Using BamTools 2.5.1^56^, ChIP replicates for each construct were merged, sorted, and a bigWig file was generated at 100 base-pair resolution excluding mm9-blacklisted regions using the bamCoverage module of deepTools. DeepTools was also used to normalize all ChIP-samples collected from Cbx.VD-expressing cells (Cbx.VD ChIP) to a GFP-control sample, plot correlation matrices, and plot average profiles around antibody-specific peaks for histone modifications. In brief, we generated a bigWig file based on two BAM files (Cbx.VD ChIP and GFP control ChIP) that were compared to each other while being simultaneously normalized for sequencing depth (using BamCompare –normalize, using reads per million mapped reads and excluding blacklisted regions). To obtain genome-wide 1-kb intervals, we partitioned the entire genome into 1-kb-sized bins. Intervals overlapping with ENCODE blacklisted regions were removed to reduce artefacts due to annotation errors. MultiBigwigSummary was used to compute the average scores for each of the bigWig files in every genomic interval. To detect antibody-specific peaks for histone modifications, previously published ChIP-seq samples^16^ were used and MACS2 was applied with input chromatin as a background signal using the following parameters: --broad, -g mm, --broad-cutoff 0.05. Finally, computeMatrix was used to calculate the coverage per defined genome regions (antibody-specific peaks for H3K27me3 and H3K9me3) and to prepare an intermediate file that could be used with plotProfiles^55^.

### Immunoblotting

Whole cell lysates (WCL) of cells stably expressing dCas9-fused repressors were prepared in 50 mM Tris-HCl, pH 7.5, 150 mM NaCl, 1 mM EDTA, 1% TritonX-100, 1 mM DTT, and cOmplete protease inhibitor cocktail (Roche). In all, 15 µg of each WCL were loaded onto a reducing SDS-PAGE and following electrophoretic separation, proteins were transferred onto Immobilion-P polyvinylidene difluoride (PVDF) membranes (Merk Millipore) using the Trans-Blot Turbo Transfer system (BioRad). Blots were blocked overnight with a Tris-HCl pH 7.5, 150 mM NaCl, 5% skimmed milk solution (TBSM) at 4 °C, with gentle agitation. Blots were incubated with the following primary antibodies: 1 µg/ml anti-CRISPR-Cas9 (clone 7A9-3A3, Abcam) and 0.44 µg/ml anti-GAPDH (clone FF26A, Abcam) in TBSM buffer containing 0.05% Tween-20 at 25 °C, with gentle agitation, for 1 h. Blots were washed three times for 10 min with TBST (Tris-HCl pH 7.5, 150 mM NaCl, 0.1% Tween-20) and incubated at 25 °C, with gentle agitation, for 1 h with the anti-mouse IgG HRP-conjugated antibody (diluted 1:1,000, clone 7076, Cell Signalling Technology). Blots were washed as described, developed using the ECL Plus substrate (Thermo Fisher) and imaged with X-ray films.

Assessment of the expression levels of Cbx7.VD-2x and Cbx2.VD-2x in mESC wild-type or mESC *Eed*-KO cells was performed by loading 10 µg of each WCL. Membranes were blocked for 1 h at 25 °C in TBSM and probed with anti-GFP rabbit polyclonal antibody (diluted 1:1,000, ab290, Abcam) and anti-Lamin B1 mouse antibody (diluted 1:2,000, sc-374015, Santa Cruz), in TBST buffer at 4 °C, with gentle agitation, overnight. Following incubation, membranes were washed three times for 10 min with TBST and incubated at 25 °C, with gentle agitation, for 1 h with anti-rabbit (NA934, Cytiva) or anti-mouse (NA931, Cytiva) IgG HRP-conjugated antibodies (both diluted 1:10,000), respectively. Blots were washed, developed, and imaged using a ChemiDoc Touch imaging system (BioRad).

For analysis of ERK and ARPC2 repression, 20 µg of each WCL of cells stably expressing dCas9-fused repressors and gRNAs were transferred onto Immobilon-P PVDF membranes as described above. Membranes were blocked for 1 h at 25 °C in TBSM and probed with the following antibodies: 0.5 µg/ml mouse anti-CRISPR-Cas9 (clone 7A9, EMD Millipore), rabbit anti-ARPC2 (diluted 1:1000, clone EPR8533, ab133315, Abcam), mouse anti-ERK1 (diluted 1:1000, clone G-8, sc-271269, Santa Cruz) and 0.44 µg/ml anti-GAPDH (clone FF26A, Abcam) in TBSM buffer containing 0.05% Tween-20 at 4 °C, with gentle agitation, overnight. Following incubation with primary antibodies, membranes were washed three times for 10 min with TBST and incubated at 25 °C, with gentle agitation, for 1 h with the anti-mouse IgG HRP-conjugated antibody (diluted 1:1000, clone 7076, Cell Signalling Technology) or the anti-rabbit IgG HRP-conjugated antibody (diluted 1:1000, clone 7074, Cell Signalling Technology). Blots were washed, developed, and imaged using X-ray films, and band intensities were quantified by densitometry using the ImageJ software (version 1.53t). Percent relative expression was calculated as 100x (band intensity of untransduced cells/ band intensity of samples stably expressing CRISPRi repressors and gRNAs).

### Reporting summary

Further information on research design is available in the Nature Research Reporting Summary linked to this article.

## Supplementary information

Supplementary Information

Description of additional Supplementary File

Supplementary Dataset 1

Supplementary Dataset 2

Supplementary Dataset 3

Supplementary Dataset 4

Supplementary Dataset 5

Supplementary Dataset 6

Reporting Summary

## Data Availability

The data that support this study are available from the corresponding authors upon reasonable request. All sequencing datasets produced in this study have been deposited to the NCBI Gene Expression Omnibus under the accession: GSE188439. Additional genomics datasets used in this study include mESC H3K4me3, H3K9me3, and H3K27me3, which can be found under the accession: GSE128907. Source data are provided with this paper.

## Source data

Source data
