# Author Correction: High-affinity chromodomains engineered for improved detection of histone methylation and enhanced CRISPR-based gene repression

**DOI:** 10.1038/s41467-022-35175-8

**Published:** 2022-11-30

**Authors:** G. Veggiani, R. Villaseñor, G. D. Martyn, J. Q. Tang, M. W. Krone, J. Gu, C. Chen, M. L. Waters, K. H. Pearce, T. Baubec, S. S. Sidhu

**Affiliations:** 1 The Anvil Institute, Kitchener, ON N2G 1H6 Canada; 2 Department of Pathobiological Sciences, School of Veterinary Medicine, Louisiana State University, Baton Rouge, LA 70803 USA; 3 Division of Molecular Biology, Biomedical Center Munich, Ludwig-Maximilians-University, 82152 Planegg-Martinsried, Germany; 4 Department of Molecular Mechanisms of Disease, University of Zurich, 8057 Zurich, Switzerland; 5 School of Pharmacy, University of Waterloo, Waterloo, ON N2L 3G1 Canada; 6 Department of Chemistry, University of North Carolina at Chapel Hill, CB 3290, Chapel Hill, NC 27599 USA; 7 Center for Integrative Chemical Biology and Drug Discovery, Division of Chemical Biology and Medicinal Chemistry, UNC Eshelman School of Pharmacy, University of North Carolina at Chapel Hill, Chapel Hill, NC 27599 USA; 8 Division of Genome Biology and Epigenetics, Institute of Biodynamics and Biocomplexity, Department of Biology, Utrecht University, 3584 Utrecht, The Netherlands

**Keywords:** Methylation analysis, Genetic engineering

Correction to: *Nature Communications* 10.1038/s41467-022-34269-7, published online 15 November 2022

In this article the author name M. W. Krone was incorrectly written as W. M. Krone. The original article has been corrected.

