# Supplementary material for: High-affinity chromodomains engineered for improved detection of histone methylation and enhanced CRISPR-based gene repression
Source: Nat Commun. 2022 Nov 15;13:6975. doi: 10.1038/s41467-022-34269-7 (PMC9666628; doi:10.1038/s41467-022-34269-7)
Supplement: Supplementary file 1 — Supplementary Information [file 41467_2022_34269_MOESM1_ESM.pdf]

Supplementary Information

High-affinity chromodomains engineered for improved detection of histone methylation and enhanced CRISPR-based gene repression

Veggiani G.[1,2,*], Villaseñor R.[3,4,†], Martyn G.D.[1,5,†],Tang J.Q.[1,5], Krone W.M.[6], Gu J.[1], Chen C.[1], Waters M.L.[6], Pearce K.H.[7], Baubec T.[4,8], Sidhu S.S.[1,5,*]

[1] The Anvil Institute, 151 Charles Street West, Kitchener, ON N2G 1H6, Canada.

[2] Department of Pathobiological Sciences, School of Veterinary Medicine, Louisiana State University, Baton Rouge, LA 70803, United States.

[3] Division of Molecular Biology, Biomedical Center Munich, Ludwig-Maximilians-University, 82152 Planegg-Martinsried, Germany.

[4] Department of Molecular Mechanisms of Disease, University of Zurich, Winterthurerstrasse 190, 8057, Zurich, Switzerland.

[5] School of Pharmacy, University of Waterloo, Waterloo, ON N2L 3G1, Canada.

[6] Department of Chemistry, University of North Carolina at Chapel Hill, CB 3290, Chapel Hill, North Carolina 27599, United States.

[7] Center for Integrative Chemical Biology and Drug Discovery, Division of Chemical Biology and Medicinal Chemistry, UNC Eshelman School of Pharmacy, University of North Carolina at Chapel Hill, Chapel Hill, North Carolina 27599, United States.

[8] Division of Genome Biology and Epigenetics, Institute of Biodynamics and Biocomplexity, Department of Biology, Utrecht University, Padualaan 8, 3584, Utrecht, The Netherlands.

†These authors contributed equally to this work.

*To whom correspondence should be addressed:
Sachdev Sidhu, sachdev.sidhu@uwaterloo.ca
Gianluca Veggiani, gveggiani@lsu.edu

	Peptide ID	Sequence
Non-modified	H3K9	ARTKQTARKSTGGKAPR
	H3K14	TARKSTGGKAPRKQLAT
	H3R17	KSTGGKAPRKQLATKAA
	H3K27	KQLATKAARKSAPATGGVK
	H3K36	KSAPSTGGVKKPHRYKPGT
	H4K20	GGAKRHRKVLRDNIQ
Methylated	H3K9Me1	ARTKQTAR**Kme1**STGGKAPR
	H3K9Me2	ARTKQTAR**Kme2**STGGKAPR
	H3K9Me3	ARTKQTAR**Kme3**STGGKAPR
	H3R17me1	KSTGGKAP**Rme1**KQLATKAA
	H3K27Me1	KQLATKAAR**Kme1**SAPATGGVK
	H3K27Me2	KQLATKAAR**Kme2**SAPATGGVK
	H3K27Me3	KQLATKAAR**Kme3**SAPATGGVK
	H3K36Me3	KSAPSTGGV**Kme3**KPHRYKPGT
	H4K20Me3	GGAKRHR**Kme3**VLRDNIQ
Acetylated	H3K14Ac	TARKSTGG**Kac**APRKQLAT
	H3K9Ac	ARTKQTAR**Kac**STGGKAPR
	H3K27Ac	KQLATKAAR**Kac**SAPATGGVK

Supplementary Table 1. Sequences of peptides used in this work. All peptides were modified at the N-terminus with biotin for immobilization onto microplates via streptavidin, or FITC for fluorescence polarization experiments. Residues carrying post-translational modifications are shown in bold and red.

Target peptide	[Cbx7.V1] (μM)	[peptide] (μM)	K_D (μM)	ΔG (kcal/mol)	ΔH (kcal/mol)	-TΔS (kcal/mol)	N (sites)
H3K9me3	70	1.2	7.9	-6.96	-8.06	1.10	1.7
	112	2	5.83	-7.14	-7.19	0.05	1.25
	112	2	7.2	-7.02	-7.6	0.58	1.27
H3K27me3	70	1.2	8.34	-6.93	-9.02	2.09	1.40
	112	2	7.63	-6.98	-7.92	0.93	0.99
	112	2	7.47	-7	-7.96	0.97	0.98

Supplementary Table 2. Thermodynamic parameters from ITC measurements for Cbx7.V1 binding to H3K9me3 and H3K27me3 peptides. Values were obtained from three independent experiments.

Oligonucleotide	Sequence
oGV-Cbx7-R1	GGCAGCAGCGCAATT(7)(7)(8) (7)(5)(5) (6)(5)(7) (7)(8)(8) (7)(6)(5)GTTGAAAGCATTCGTAAAAAACG
oGV-Cbx7-R2	CGTAAAGGTAAAGTTGAATATCTGGTTAAA(8)(7)G(5)(5)A(7)(7)(8)(8)(7)G(6)(6)G(6) (6)G(5)(5)A(8)(5)(8)(5)(7)(6)(5)(6)(6)TGGGAACCGGAAGAACATATTCTG

Numbers indicate nucleotide mixtures of 70% of wild-type nucleotide (represented by 5=A, 6=C, 7=G and 8=T) and 10% of each of the other three nucleotides.

Supplementary Table 3. Sequences of mutagenic oligonucleotides for construction of the phage-displayed Cbx7.V library.

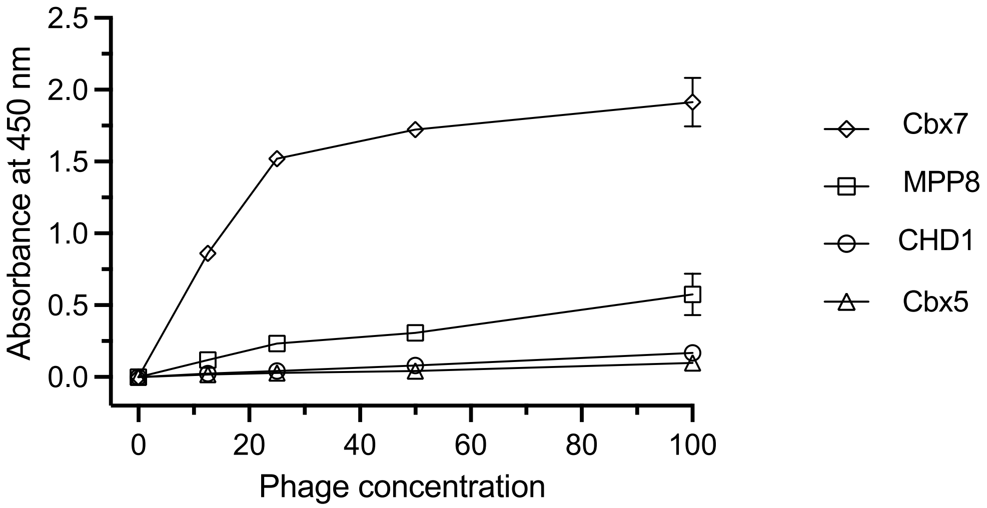

Supplementary Figure 1. Phage ELISA binding curves of different chromodomains to an immobilized anti-Flag tag antibody (mean of triplicate ±1 SD).

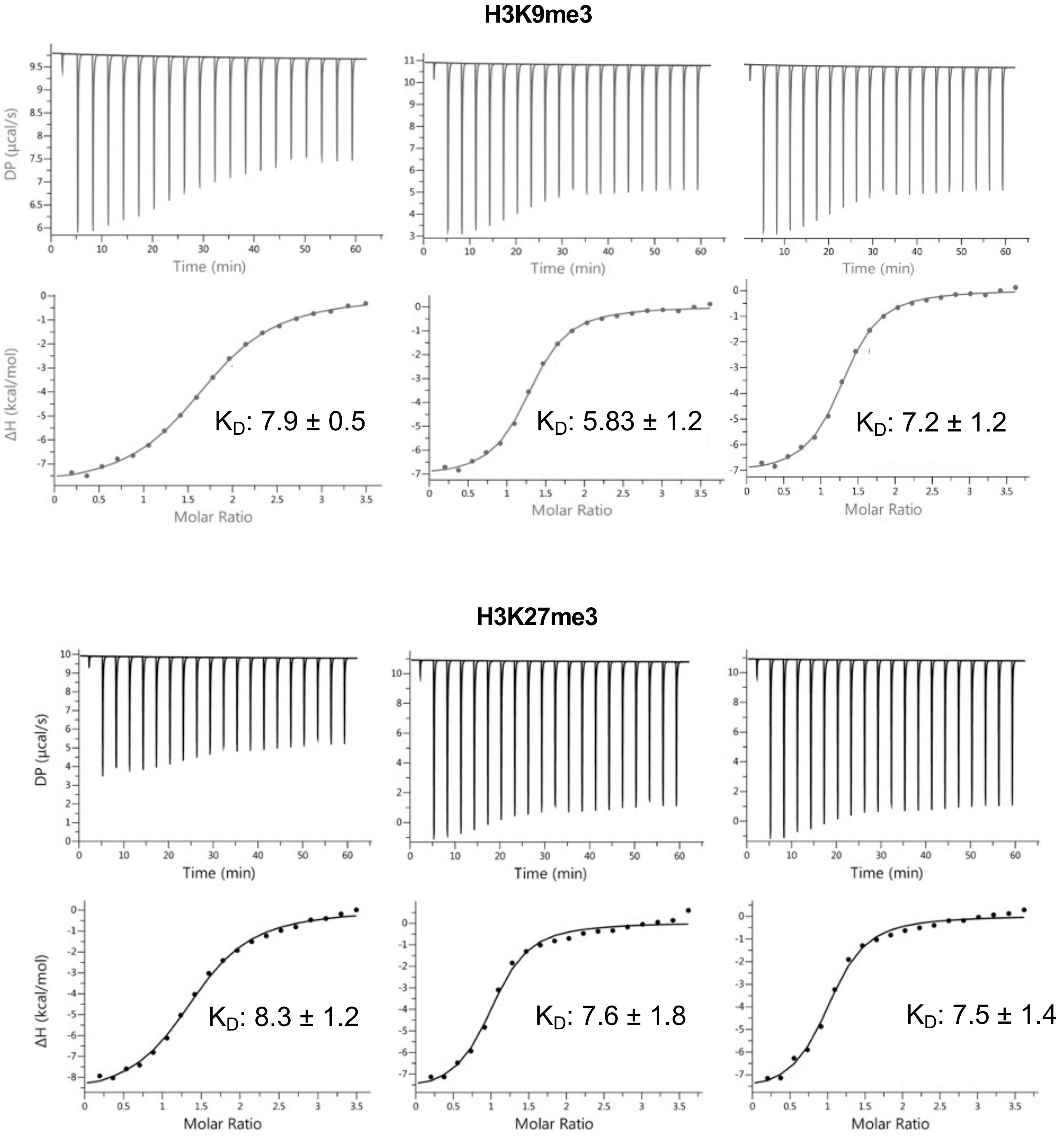

Suppelemtary Figure 2. Representative ITC isotherms for Cbx7.V1 binding to H3K9me3 or H3K27me3 peptide. Dissociation constants (K_D) were determined from three independent experiments. See **Supplementary Table 2** for thermodynamic parameters.

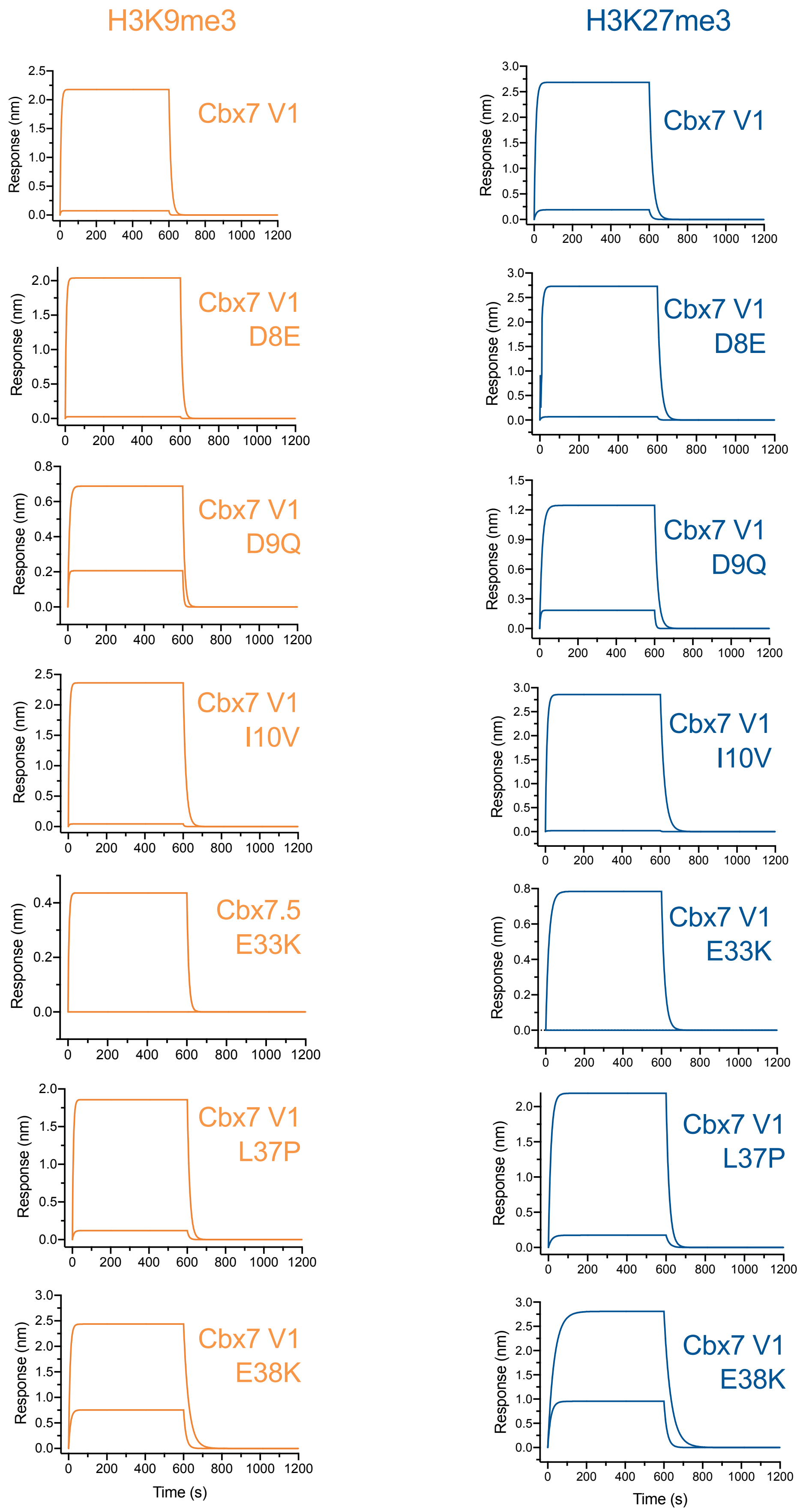

Supplementary Figure 3. Representative sensograms from binding of Cbx7.V1 variants to immobilized biotinylated H3K9me3 or H3K27me3 peptide.

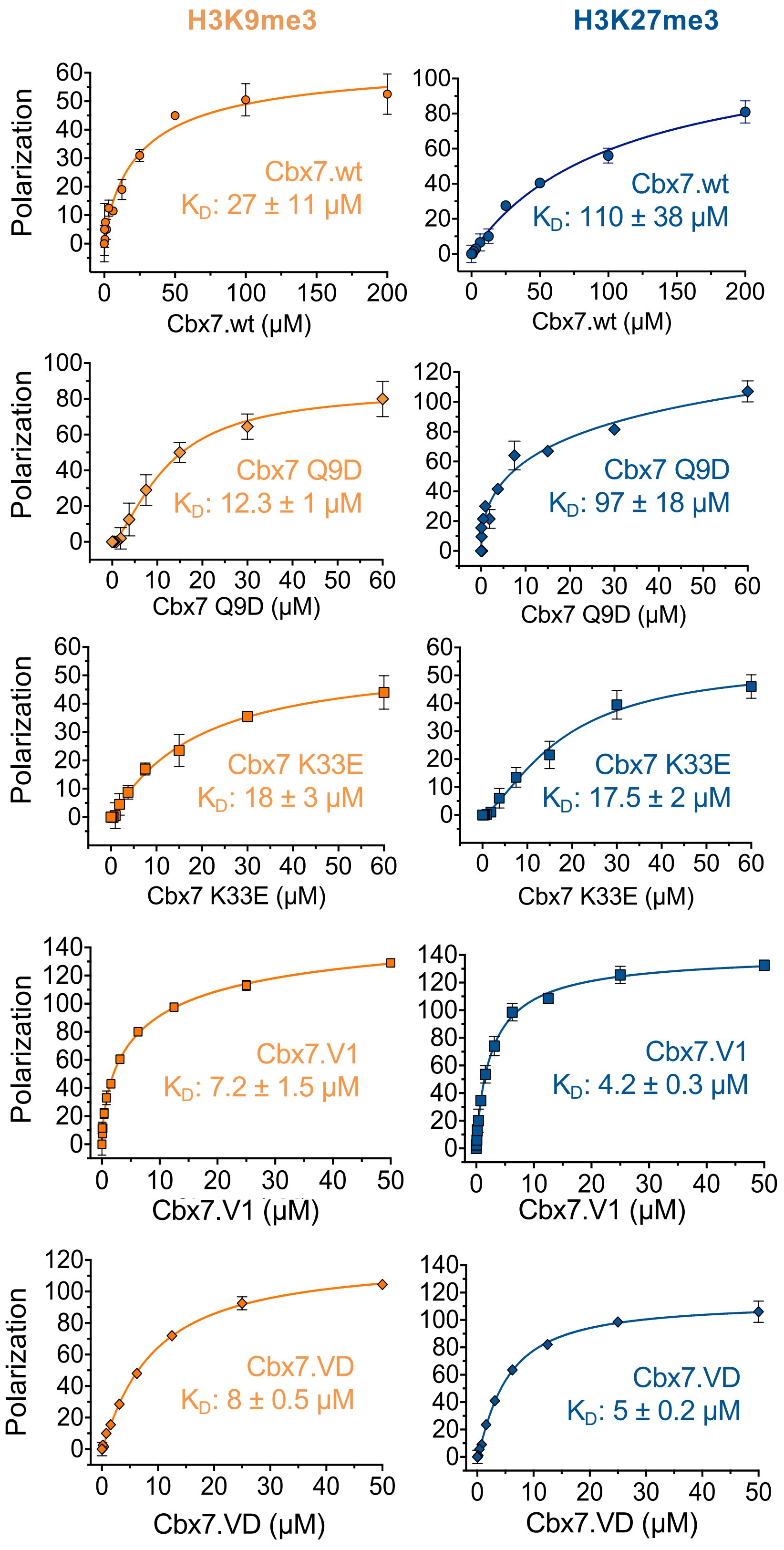

Supplementary Figure 4. Fluorescence polarization binding curves of Cbx7.wt and variants binding to H3K9me3 or H3K27me3 peptide. Varying concentrations of chromodomain were titrated against FITC-labelled peptides. Data represent the mean and standard deviation of triplicate measurements.

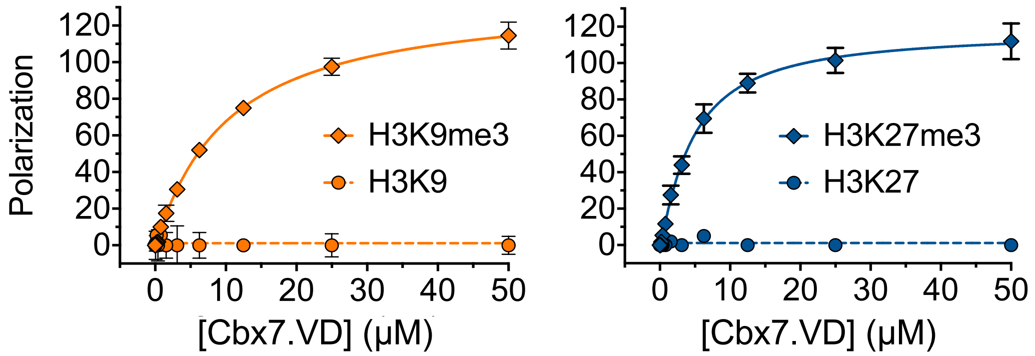

Supplementary Figure 5. Binding of Cbx7.VD to H3K9 and H3K27 methylated (solid line) or unmethylated (dotted lines) peptides was determined by fluorescence polarization. Varying concentrations of Cbx7.VD (50 µM – 50 nM) were titrated against FITC-labelled peptides and polarization values were measured at 30 minutes (mean of triplicate ±1 SD).

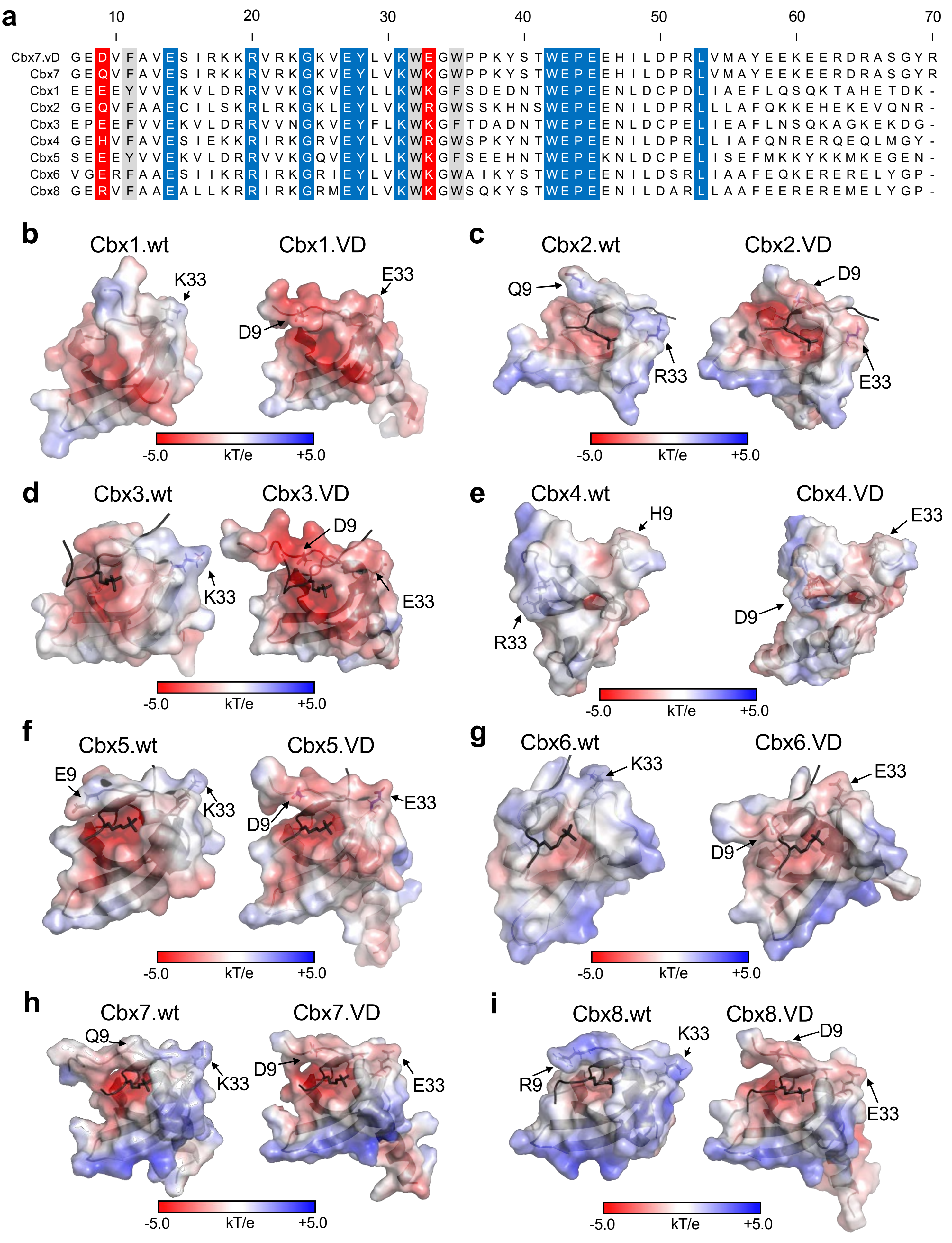

(legend on next page)

Supplementary Figure 6. (**a**) Sequence alignment of all chromodomains of Cbx proteins and Cbx7.VD. Numbering was based on full length Cbx7.wt, conserved residues are shaded in blue, whereas positions that were mutated as in Cbx7.VD are shaded in red. Amino acids forming the aromatic cage are shaded in grey. (**b-i**) Electrostatic surface potential of wild-type Cbx chromodomains and Cbx.VD models whose structure prediction was performed in the ColabFold Notebook-based environment[30]. The electrostatic potentials were calculated using the non-linear Poisson-Boltzmann equation contoured at 5 kT/e with PyMol and the APBS plugin (PyMOL Molecular Graphics System, Version 2.0 Schrödinger, LLC). Negatively and positively charged surfaces are coloured in red and blue, respectively. Positions 9 and 33 in chromodomains are represented as sticks. Methylated peptides are coloured black, with the methyllysine side chain shown as sticks.

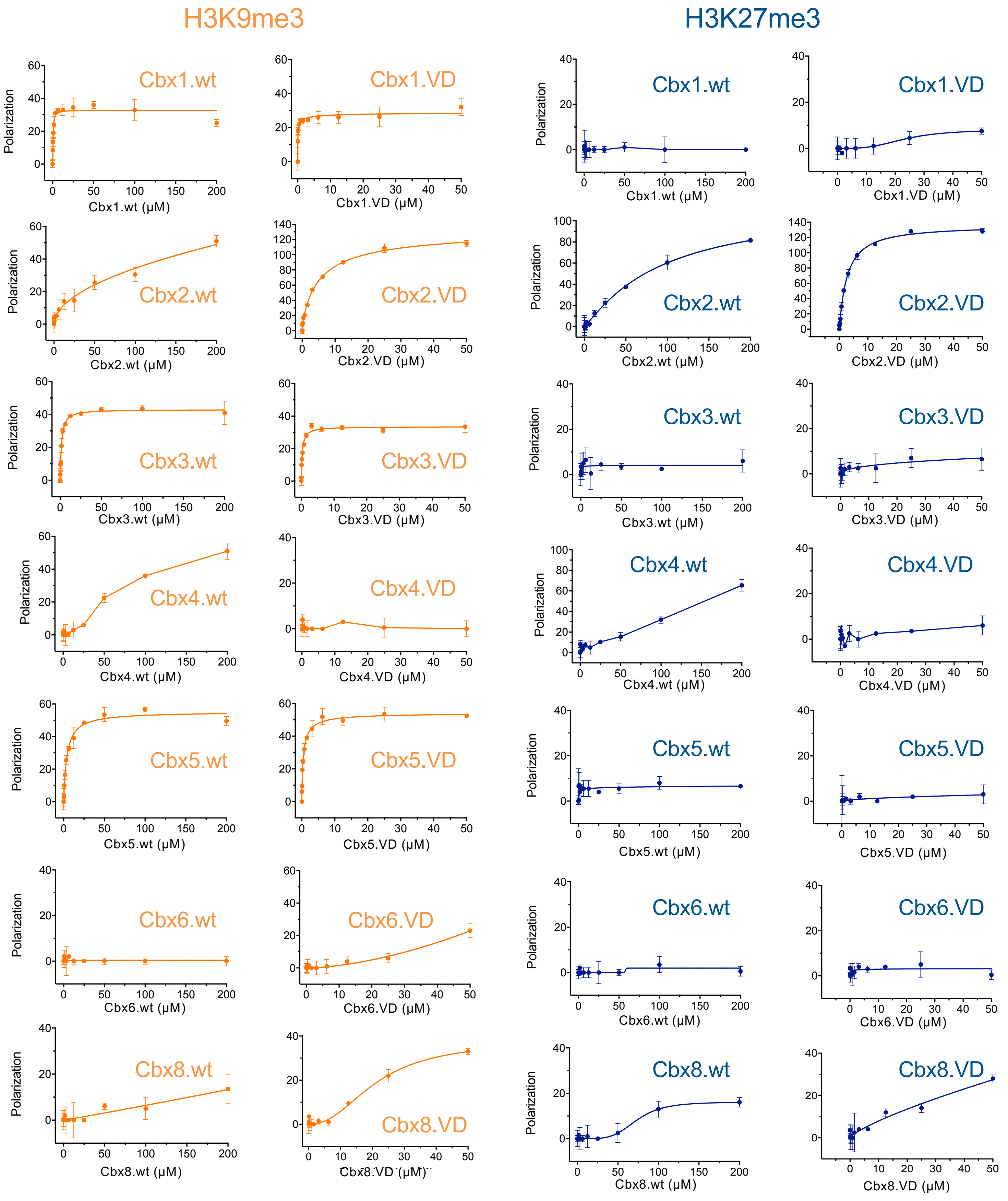

Supplementary Figure 7. Fluorescence polarization binding curves of all Cbx.wt and Cbx.VD pairs binding to H3K9me3 or H3K27me3 peptide. Varying concentrations of chromodomain were titrated against FITC-labelled peptide. Data represent the mean and standard deviation of triplicate measurements.

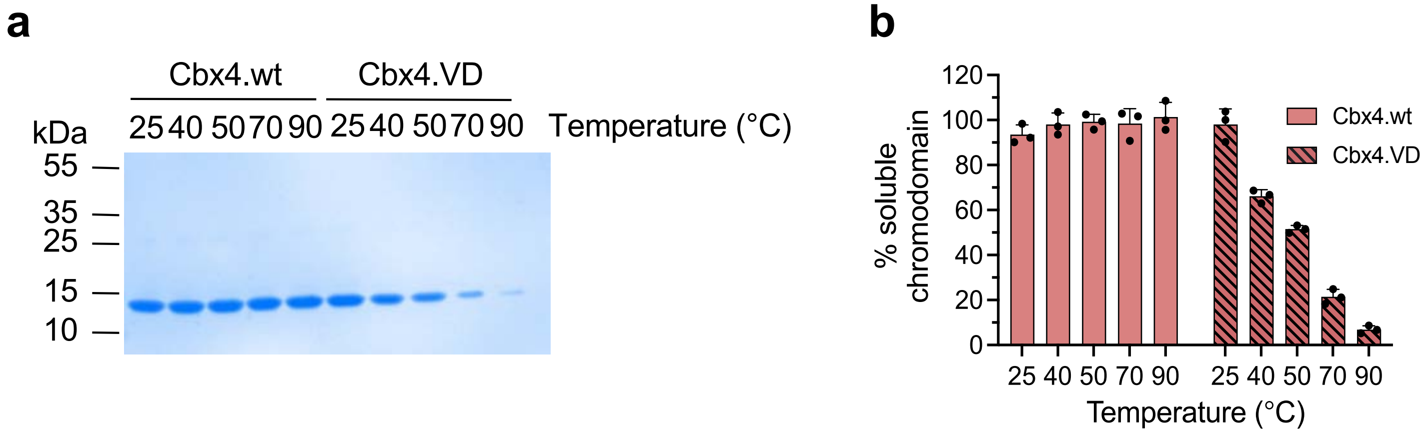

Supplementary Figure 8. Thermostability of Cbx4.wt and Cbx4.VD. **a.** A representative SDS-PAGE showing decreased thermostability of Cbx4.VD. Chromodomains were heated at the indicated temperature for 3 min, centrifuged to remove aggregates, and analyzed by SDS-PAGE with Coomassie staining. Similar results were obtained from three independent experiments. **b.** Histogram shows the % of soluble chromodomain determined by densitometry. Data were analyzed from three independent experiments and expressed as mean ± SD.

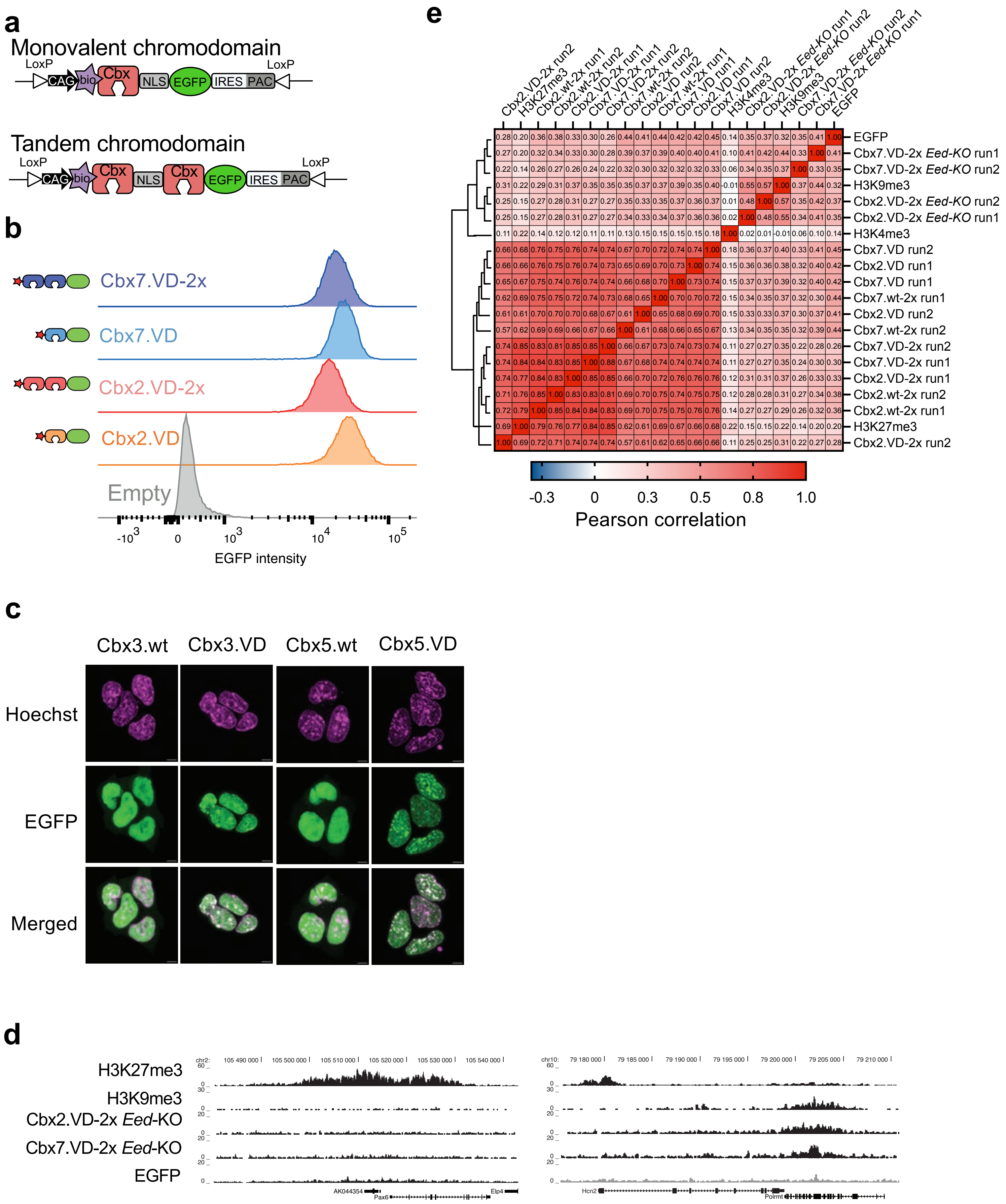

(continued on next page)

f

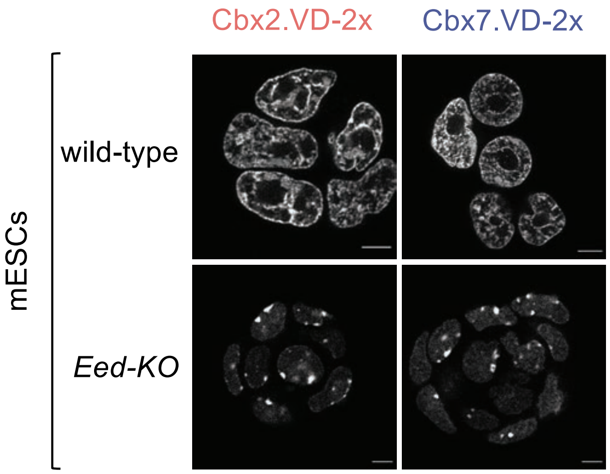

g

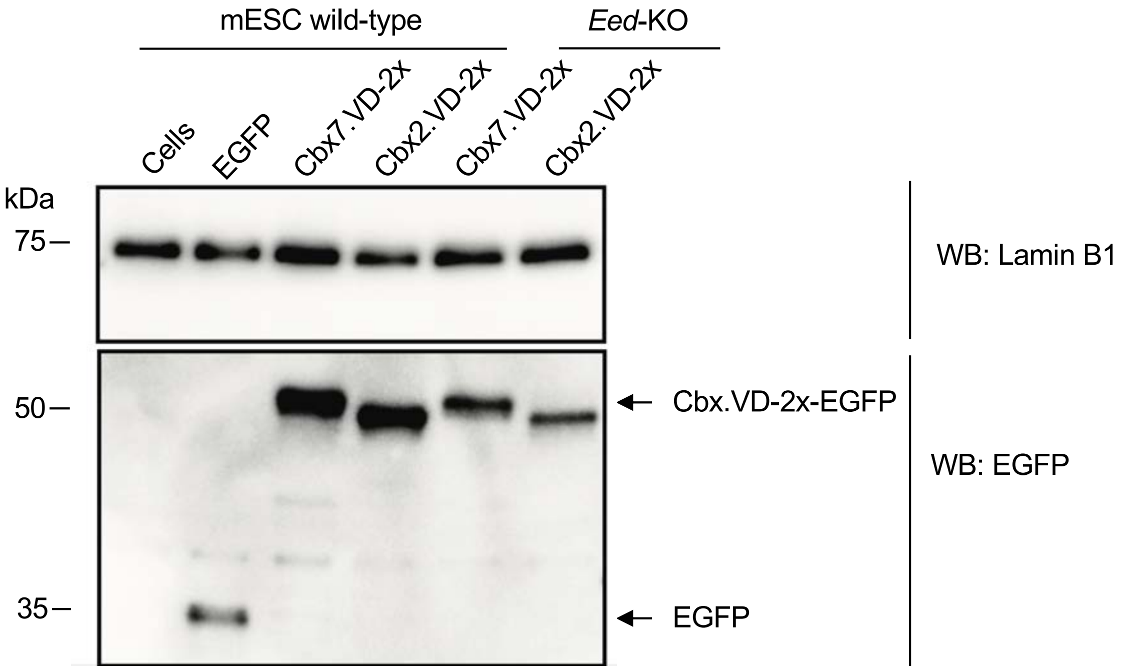

Supplementary Figure 9. (**a**) Schematics of the constructs used to obtain mESCs stably expressing engineered chromodomains. Cbx.VDs are composed of single or double chromodomains fused in frame to EGFP for live-cell imaging experiments. At the N-terminus constructs were fused to a biotin acceptor site for site-specific biotinylation and biotin ChiP-seq experiments. (**b**) Flow cytometry profiles of Cbx.wt and Cbx.VDs fused to EGFP stably integrated into mESCs. (**c**) Live-cell imaging of mESCs stably expressing Cbx3.wt, Cbx3.VD, Cbx5.wt and Cbx5.VD chromodomains fused to EGFP. Data show representative confocal images from two independent experiments. Scale bar 5 µm. (**d**) Genome browser examples for loss of binding of engineered chromodomains to H3K27me3 sites. (**e**) Correlation analysis showing genome-wide interaction of Cbx.VDs with histone modifications. Pearson correlation score was calculated based on counts at 1 kb intervals. (**f**) Live-cell imaging showing nuclear localization of Cbx2.VD-2x and Cbx7.VD-2x in mESC and *Eed-KO* cells. Similar results were obtained from two independent experiments. Scale bar 5 µm. (**g**) Comparison of the expression levels of Cbx2.VD-2x and Cbx7.VD-2x in mESC and *Eed*-KO cells. The expression of each chromodomain was assessed using an anti-EGFP antibody, whereas equal loading was detected with an anti-Lamin B1 antibody. Similar results were obtained from two independent experiments.

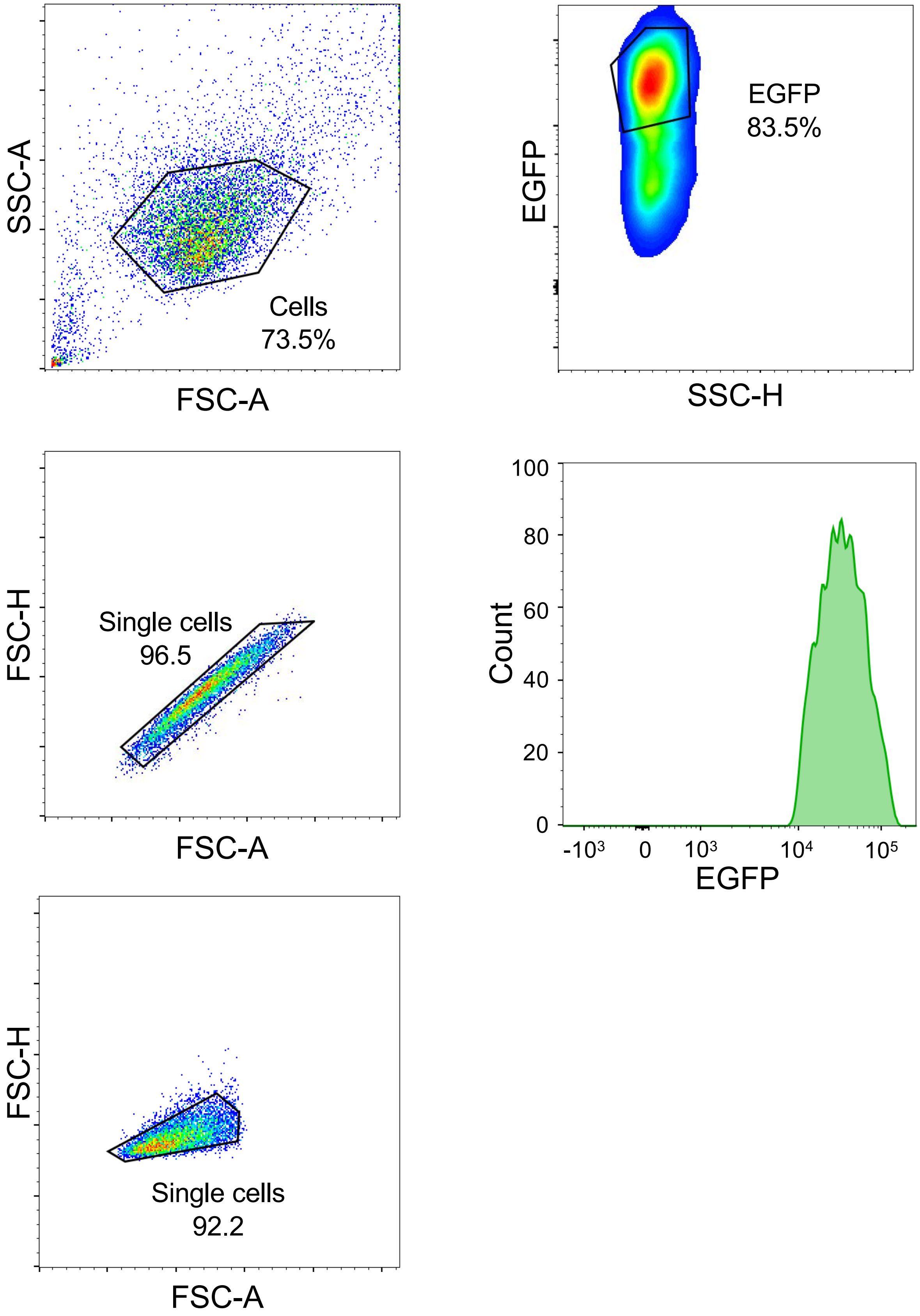

Supplementary Figure 10. Example of the gating strategy for flow cytometry experiments. Samples were first gated for single cells and then for EGFP expression. The geometric mean of EGFP fluorescence was calculated from the final gated population. An identical gating strategy was used for the assessment of CD81 cell surface levels upon CRISPRi (see Figure 5b).

a

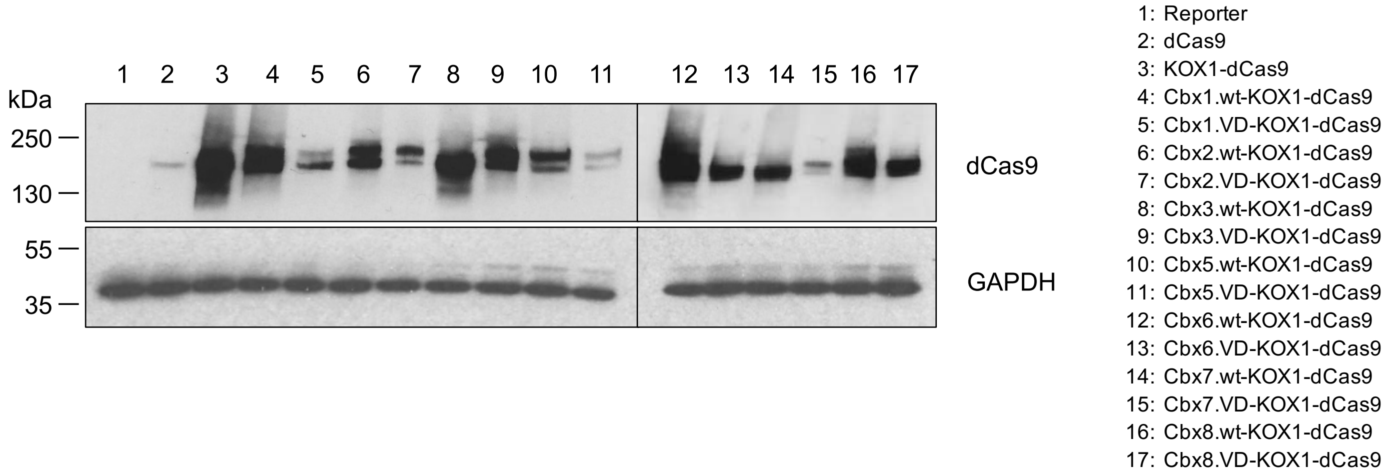

b

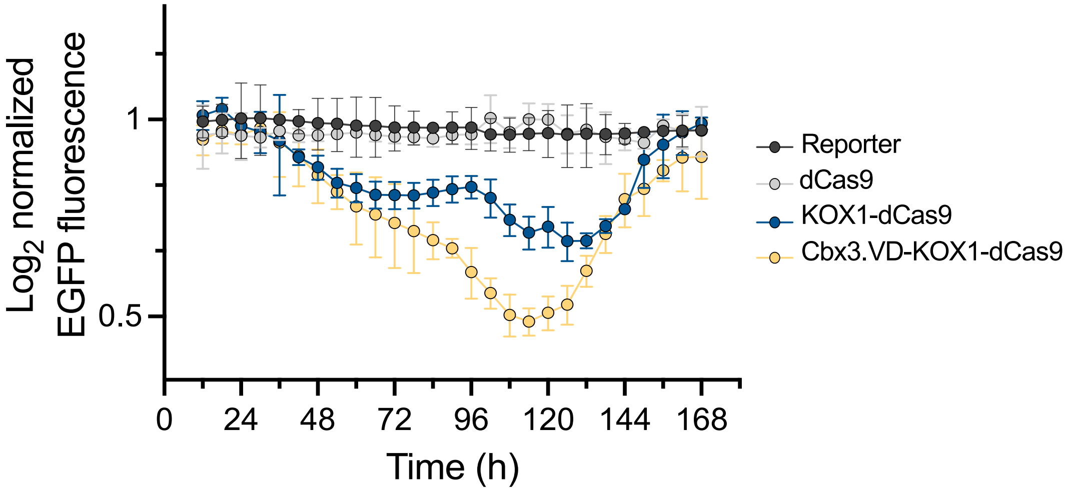

Supplementary Figure 11. (**a**) Assessment of the expression levels of different KRAB-dCas9 fusion proteins. Expression of repressors was assayed using an anti-Cas9 antibody. Equal loading was detected through an anti-GAPDH antibody. Data show a representative western blot from three independent experiments. (**b**) HEK293T-SV40 EGFP reporter cells were seeded into 96-well plates and transiently transfected with plasmids encoding dCas9, KOX1-dCas9 or Cbx3.VD-KOX1-dCas9. 12 hours after transfection the fluorescence intensity of EGFP was monitored every 6 hours for one week. Following ectopic expression of repressor genes, EGFP levels decreased and reverted to baseline levels as a consequence of cell division and plasmid loss. Fluorescence intensity from three independent experiments was normalized against the number of cells present in each well and expressed as mean ± SD.

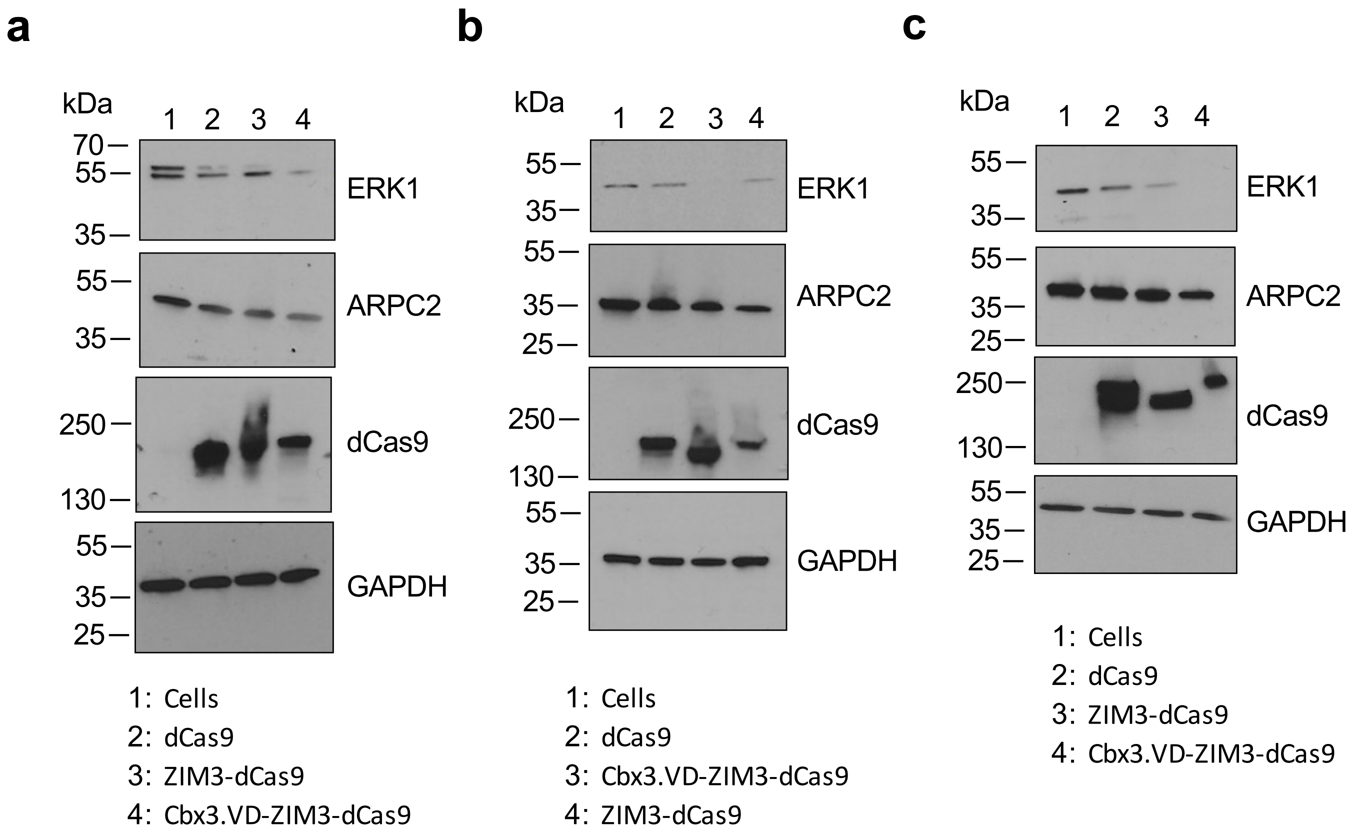

Supplementary Figure 12. Representative western blots for analysis of the expression levels of ERK1 and ARPC2 proteins upon CRISPRi. HEK293T (**a**), U20S (**b**) and HeLa (**c**) cells were transduced with different CRISPRi repressors and gRNAs targeting the *ERK1* and *ARPC2* TSS. Two guides per gene were used, and the expression levels of each targeted protein and of each dCas9-fusion were assayed using an anti-ERK1, anti-ARPC2 and anti-Cas9 antibody, respectively. Equal loading was determined using an anti-GAPDH antibody. Similar results were obtained from two independent experiments.

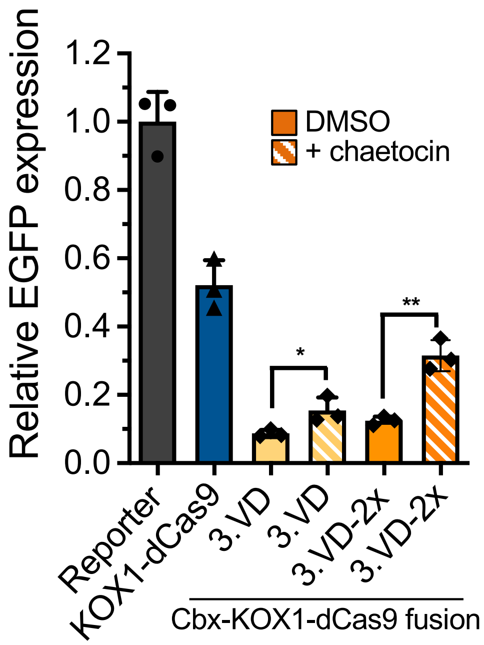

Supplementary Figure 13. Comparison of the repression by KOX1-dCas9 fused to single (Cbx3.VD) or double (Cbx3.VD-2x) chromodomains. EGFP fluorescence signals were measured by flow cytometry following cell treatment for 24 hours with 10 µM of the histone lysine methyltransferase inhibitor chaetocin (striped bars) or DMSO (filled bars). Data are mean of three independent experiments ± 1 SD. Asterisks denote *p* values (two-tailed unpaired t-test, * $p = 0.0161$, ** $p = 0.0092$).
