# Supplementary material for: High-affinity chromodomains engineered for improved detection of histone methylation and enhanced CRISPR-based gene repression
Source: Nat Commun. 2022 Nov 15;13:6975. doi: 10.1038/s41467-022-34269-7 (PMC9666628; doi:10.1038/s41467-022-34269-7)
Supplement: Supplementary file 2 — Description of additional Supplementary File [file 41467_2022_34269_MOESM2_ESM.pdf]

Description of Additional Supplementary files

Supplementary Datasets

**Supplementary Dataset 1**	PDB accession codes and hyperlinks of crystal structures analyzed in this study
**Supplementary Dataset 2**	Chromodomain boundaries used in the study
**Supplementary Dataset 3**	Sequences of wt Cbx chromodomains and Cbx.VDs cloned into pET21b vector for expression in *E.Coli*
**Supplementary Dataset 4**	Sequences of chromodomains used in live-cells imaging and ChIP experiments
**Supplementary Dataset 5**	Sequences of all dCas9-based repressors used in the study
**Supplementary Dataset 6**	Sequences of gRNAs used in the study
