# Supplementary material for: High-affinity chromodomains engineered for improved detection of histone methylation and enhanced CRISPR-based gene repression
Source: Nat Commun. 2022 Nov 15;13:6975. doi: 10.1038/s41467-022-34269-7 (PMC9666628; doi:10.1038/s41467-022-34269-7)
Supplement: Supplementary file 9 — Reporting Summary [file 41467_2022_34269_MOESM9_ESM.pdf]

nature portfolio

Corresponding author(s): Sachdev Sidhu, Gianluca Veggiani

Last updated by author(s): Sep 14, 2022

Reporting Summary

Nature Portfolio wishes to improve the reproducibility of the work that we publish. This form provides structure for consistency and transparency in reporting. For further information on Nature Portfolio policies, see our Editorial Policies and the Editorial Policy Checklist.

Statistics

For all statistical analyses, confirm that the following items are present in the figure legend, table legend, main text, or Methods section.

n/a	Confirmed	
☐	☒	The exact sample size (*n*) for each experimental group/condition, given as a discrete number and unit of measurement
☐	☒	A statement on whether measurements were taken from distinct samples or whether the same sample was measured repeatedly
☐	☒	The statistical test(s) used AND whether they are one- or two-sided<br>*Only common tests should be described solely by name; describe more complex techniques in the Methods section.*
☒	☐	A description of all covariates tested
☐	☒	A description of any assumptions or corrections, such as tests of normality and adjustment for multiple comparisons
☐	☒	A full description of the statistical parameters including central tendency (e.g. means) or other basic estimates (e.g. regression coefficient) AND variation (e.g. standard deviation) or associated estimates of uncertainty (e.g. confidence intervals)
☐	☒	For null hypothesis testing, the test statistic (e.g. *F*, *t*, *r*) with confidence intervals, effect sizes, degrees of freedom and *P* value noted<br>*Give P values as exact values whenever suitable.*
☒	☐	For Bayesian analysis, information on the choice of priors and Markov chain Monte Carlo settings
☒	☐	For hierarchical and complex designs, identification of the appropriate level for tests and full reporting of outcomes
☐	☒	Estimates of effect sizes (e.g. Cohen's *d*, Pearson's *r*), indicating how they were calculated

Our web collection on statistics for biologists contains articles on many of the points above.

Software and code

Policy information about availability of computer code

Data collection	ELISA measurements were performed on a Biotek Powerwave XS microplate reader, whereas luminescence was collected with the Biotek Synergy 5 plate reader. Fluorescence polarization data were collected with the Synergy Neo2 multi-mode assay plate reader (Biotek), and bio-layer interferometry measurements were collected on the Fortebio Octet HTX system. Isothermal calorimetry data were collected with the MicroCal PEAQ-ITC automated calorimeter (version 1.1.0.1262, Malvern Instruments). Expression levels of Cbx-EGFP fusions stably integrated into mESC genome were determined by flow cytometry on a BD Biosciences FACSCanto. For CRISPRi experiments cells were analyzed on a BD Bioscience LSR Fortessa cytometer. Confocal microscopy was performed on a Leica SP5 microscope.
Data analysis	ELISA, luminescence and dissociation constants were analyzed using Prism v.9.3. Flow cytometry data were analyzed using FlowJo v.10.8, whereas confocal images were analyzed with Fiji 2.0.0rc-68/1.52e. Densitometry data were analyzed with ImageJ software v1.53t (for western blot experiments) and with the Image Lab 3.0 software (Bio-Rad) for Cbx4.VD thermostability analysis. Isothermal calorimetry data were analyzed with MicroCal PEAQ-ITC analysis software (version 1.1.0.1262, Malvern Instruments).<br>ChIP data were analyzed using:<br>R version 3.5.2, trim_galore v.0.4.3, Bowtie2 v.2.3.5.1, samtools v.1.11, Picard tools v.2.23.9, deeptools v.3.5, bamtools v.2.5.1.

For manuscripts utilizing custom algorithms or software that are central to the research but not yet described in published literature, software must be made available to editors and reviewers. We strongly encourage code deposition in a community repository (e.g. GitHub). See the Nature Portfolio guidelines for submitting code & software for further information.

Data

Policy information about availability of data

All manuscripts must include a data availability statement. This statement should provide the following information, where applicable:

- Accession codes, unique identifiers, or web links for publicly available datasets
- A description of any restrictions on data availability
- For clinical datasets or third party data, please ensure that the statement adheres to our policy

All data generated are available in the Source data file. Other data supporting the findings of this study are available from the authors upon reasonable request. All sequencing datasets produced in this study have been deposited to the NCBI Gene Expression Omnibus under the accession: GSE188439. Additional genomics datasets used in this study include mESC H3K4me3, H3K9me3, and H3K27me3 (GSE128907). To review GEO accession GSE188439 go to https://www.ncbi.nlm.nih.gov/geo/query/acc.cgi?acc=GSE188439, and enter the uryngsgcvjghvqn token into the box. All plasmids are available from the authors upon request.

Accession codes of analyzed crystal structures are Cbx1, PDB: 3F2U; Cbx2, PDB: 3H91; Cbx3, PDB: 3DM1; Cbx4, PDB: 5EPL; Cbx5, PDB: 3FDT; Cbx6, PDB: 3I90; Cbx7, PDB: 4X3K; Cbx8, PDB: 3I91. Hyperlinks to each deposited structure are available in Supplementary File 1.

Field-specific reporting

Please select the one below that is the best fit for your research. If you are not sure, read the appropriate sections before making your selection.

[x] Life sciences [ ] Behavioural & social sciences [ ] Ecological, evolutionary & environmental sciences

For a reference copy of the document with all sections, see nature.com/documents/nr-reporting-summary-flat.pdf

Life sciences study design

All studies must disclose on these points even when the disclosure is negative.

Sample size	Sample sizes were not predetermined and were based on previous studies (ref. 16, 38). We performed three replicates for most experiments to ensure data reproducibility and robustness.
Data exclusions	No data were excluded from analysis
Replication	All replicates are included in the manuscript. All attempts at replication were successful. Most experiments were performed in three replicates unless otherwise noted in the manuscript. Statistical analysis was conducted on data from three or more biologically independent experimental replicates. Error bars displayed on graphs represent the mean ± SD of at least three independent experiments. Statistical significance was analyzed using two-sided unpaired student's t test, with $p<0.05$ considered significant.
Randomization	No randomization was necessary. Side-by-side comparisons of binding affinity, cellular localization, ChIP performance and dCas9 fusions were tested at the same time to minimize variation between sample conditions. Randomization was not necessary as this study does not involve animals or human participants.
Blinding	Samples were not blinded as this study does not involve animals or human participants.

Reporting for specific materials, systems and methods

We require information from authors about some types of materials, experimental systems and methods used in many studies. Here, indicate whether each material, system or method listed is relevant to your study. If you are not sure if a list item applies to your research, read the appropriate section before selecting a response.

Materials & experimental systems

n/a	Involved in the study	
[ ]	[x]	Antibodies
[ ]	[x]	Eukaryotic cell lines
[x]	[ ]	Palaeontology and archaeology
[x]	[ ]	Animals and other organisms
[x]	[ ]	Human research participants
[x]	[ ]	Clinical data
[x]	[ ]	Dual use research of concern

Methods

n/a	Involved in the study	
[ ]	[x]	ChIP-seq
[ ]	[x]	Flow cytometry
[x]	[ ]	MRI-based neuroimaging

Antibodies

Antibodies used	The following antibodies were used in the study:<br>ELISA:<br>Monoclonal anti–M13-HRP conjugated antibody diluted 1:3,000 (SinoBiological, cat# 11973-MM05T-H), monoclonal anti-FLAG M2 antibody at 2µg/ml (Sigma, cat# F3165).<br><br>Western Blotting:<br>anti-CRISPR-Cas9 at 1 µg/ml (clone 7A9-3A3, Abcam, cat# ab191468),<br>anti-GAPDH at 0.44 µg/ml (clone FF26A, Abcam, cat# ab59164),<br>anti-mouse IgG HRP-conjugated antibody diluted 1:1,000 (clone 7076, Cell Signalling Technology, cat# 7076),<br>anti-mouse Lamin B1 (diluted 1:2000, sc-374015, Santa Cruz)<br>anti-GFP (diluted 1:1000, ab290, Abcam)<br>anti-rabbit IgG HRP-conjugated (diluted 1:10,000, NA934, Cytiva)<br>anti-mouse IgG HRP-conjugated (diluted 1:10,000, NA931, Cytiva)<br>anti-human ARPC2 (diluted 1:1,000, clone EPR8533, ab133315, Abcam)<br>anti-human ERK1 (diluted 1:1,000, clone G-8, sc-271269, Santa Cruz)<br><br>Flow cytometry:<br>PE-conjugated mouse anti-human CD81 antibody (undiluted, clone JS-81, BD Biosciences, cat# 561957).
Validation	Antibodies were purchased based on the information available on the supplier's website and on their suitability for each application (flow cytometry, western blotting). All bands in western blots were observed at expected sizes, and thus the antibodies were not further validated.<br><br>anti-CRISPR-Cas9 https://www.abcam.com/crispr-cas9-antibody-7a9-3a3-ab191468.html<br>anti-GAPDH https://www.abcam.com/gapdh-antibody-ff26a-ab59164.html<br>anti-mouse IgG HRP-conjugated antibody https://www.cellsignal.com/products/secondary-antibodies/anti-mouse-igg-hrp-linked-antibody/7076<br>anti-mouse Lamin B1 https://www.scbt.com/p/lamin-b1-antibody-b-10<br>anti-GFP https://www.abcam.com/gfp-antibody-ab290.html<br>anti-rabbit IgG HRP-conjugated https://www.sigmaaldrich.com/IT/it/product/sigma/gena9341ml<br>anti-mouse IgG HRP-conjugated https://www.sigmaaldrich.com/IT/it/product/sigma/gena9311ml<br>anti-human ARPC2 https://www.abcam.com/arpc2-antibody-epr8533-ab133315.html<br>anti-human ERK1https://www.scbt.com/p/erk-1-antibody-g-8<br>PE-conjugated mouse anti-human CD81 antibody https://www.bdbiosciences.com/ko-kr/products/reagents/flow-cytometry-reagents/research-reagents/single-color-antibodies-ruo/PE-Mouse-Anti-Human-CD81.561957

Eukaryotic cell lines

Policy information about cell lines

Cell line source(s)	HEK293T cells with stably integrated SV40-EGFP were a gift from Mikko Taipale's lab (University of Toronto). Mouse embryonic stem cells (mESCs) were obtained from Baubec et al., Cell 2015. mESC manipulations were performed using RCME as outlined in the methods. HEK293T, HeLa and U20S cells were purchased from ATCC. The Eed-KO cell line was generated as described in ref 16.
Authentication	Cell lines were not authenticated
Mycoplasma contamination	All cell lines were routinely tested for mycoplasma contamination. No tests were positive.
Commonly misidentified lines<br>(See ICLAC register)	No commonly misidentified cell lines were used.

ChIP-seq

Data deposition

- [x] Confirm that both raw and final processed data have been deposited in a public database such as GEO.
- [x] Confirm that you have deposited or provided access to graph files (e.g. BED files) for the called peaks.

Data access links<br>*May remain private before publication.*	To review GEO accession GSE188439 go to https://www.ncbi.nlm.nih.gov/geo/query/acc.cgi?acc=GSE188439, and enter the uryngsgcvjghvqn token into the box.
Files in database submission	GSM5683078 MmES_EGFP<br>GSM5683079 MmES_Cbx2.wt-2x_run1

GSM5683080 MmES_Cbx2.wt-2x_run2
GSM5683081 MmES_Cbx7.wt-2x_run1
GSM5683082 MmES_Cbx7.wt-2x_run2
GSM5683083 MmES_Cbx2.VD_run1
GSM5683084 MmES_Cbx2.VD_run2
GSM5683085 MmES_Cbx2.VD-2x_run1
GSM5683086 MmES_Cbx2.VD-2x_run2
GSM5683087 MmES_Cbx7.VD_run1
GSM5683088 MmES_Cbx7.VD_run2
GSM5683089 MmES_Cbx7.VD-2x_run1
GSM5683090 MmES_Cbx7.VD-2x_run2
GSM5683091 MmES_Cbx2.VD-2x_Eed-KO_run1
GSM5683092 MmES_Cbx2.VD-2x_Eed-KO_run2
GSM5683093 MmES_Cbx7.VD-2x_Eed-KO_run1
GSM5683094 MmES_Cbx7.VD-2x_Eed-KO_run2

Genome browser session
(e.g. UCSC)

No longer applicable

Methodology

Replicates

Two replicates per samples were performed

Sequencing depth

MmES_Cbx2.VD-2x_Eed-KO_run1.fastq.gz 29707101
MmES_Cbx2.VD-2x_Eed-KO_run2.fastq.gz 41304115
MmES_Cbx2.VD-2x_run1.fastq.gz 38617478
MmES_Cbx2.VD-2x_run2.fastq.gz 10382234
MmES_Cbx2.VD_run1.fastq.gz 34006390
MmES_Cbx2.VD_run2.fastq.gz 21354053
MmES_Cbx2.wt-2x_run1.fastq.gz 36702020
MmES_Cbx2.wt-2x_run2.fastq.gz 40052103
MmES_Cbx7.VD-2x_Eed-KO_run1.fastq.gz 27811814
MmES_Cbx7.VD-2x_Eed-KO_run2.fastq.gz 37980856
MmES_Cbx7.VD-2x_run1.fastq.gz 36414311
MmES_Cbx7.VD-2x_run2.fastq.gz 51288895
MmES_Cbx7.VD_run1.fastq.gz 38689357
MmES_Cbx7.VD_run2.fastq.gz 42756243
MmES_Cbx7.wt-2x_run1.fastq.gz 36620615
MmES_Cbx7.wt-2x_run2.fastq.gz 39907515
MmES_EGFP_run1.fastq.gz 31718368

Antibodies

Streptavidin-coated M280 magnetic beads (ThermoFisher, cat# 112050)

Peak calling parameters

macs2 callpeak -t foreground.bed -c background.bed --broad -g mm --broad-cutoff 0.01.

Data quality

Peaks on "_random chr" were excluded. Enrichments under peak regions were recalculated to confirm signals for each sample individually.

Software

ChIP data were analyzed using:
Trim_galore v.0.4.3, Bowtie2 v.2.3.5.1, samtools v.1.11, Picard tools v.2.23.9, deeptools v.3.5.

Flow Cytometry

Plots

Confirm that:

- [x] The axis labels state the marker and fluorochrome used (e.g. CD4-FITC).
- [x] The axis scales are clearly visible. Include numbers along axes only for bottom left plot of group (a 'group' is an analysis of identical markers).
- [x] All plots are contour plots with outliers or pseudocolor plots.
- [x] A numerical value for number of cells or percentage (with statistics) is provided.

Methodology

Sample preparation

To determine expression levels of Cbx-EGFP fusions integrated into mESC genome, cells were resuspended in 1x DPBS and stained with LIVE/DEAD Fixable Near-IR Dead Cell Stain (Invitrogen, L34975) to assess cell viability. Cells were analyzed on a FACSCanto flow cytometer (BD Biosciences) using the EGFP (Alexa Fluor 488-A), and live/dead (APC-Cy7A) filters to detect live

	cells expressing EGFP. For CRISPRi experiments, cells were harvested by trypsinization, washed once with ice-cold 1x DPBS, and resuspended in PBS pH 7.4 containing 1% BSA and 0.1% NaN3 (FACS-A buffer). Cells were analyzed on a LSR Fortessa X20 flow cytometer (BD Biosciences).
Instrument	FACSCanto and LSR Fortessa X20 flow cytometers (BD Biosciences)
Software	BD FACSDiva Software (BD Biosciences)
Cell population abundance	Stable cell lines generated by recombination (RCME) were confirmed to homogenously express Cbx-EGFP fusions as described below.
Gating strategy	For SV40-EGFP cells, single cells with high EBFP2 were gated. For RCME mESCs viable and individual cells were gated.

☒ Tick this box to confirm that a figure exemplifying the gating strategy is provided in the Supplementary Information.
